# From Chemistry to Pharmacology: Exploring the Anti-Inflammatory and Antioxidant Potential of Novel Dexketoprofen Amide Derivatives

**DOI:** 10.3390/antiox14070796

**Published:** 2025-06-27

**Authors:** Marko Karović, Miloš Nikolić, Nikola Nedeljković, Marina Vesović, Marina Nikolić, Marijana Anđić, Nevena Lazarević, Vladimir Jakovljević, Jelena Nedeljković, Sanja Đaković, Jelena Bošković, Vladimir Dobričić

**Affiliations:** 1 Department of Pharmacy, Faculty of Medical Sciences, University of Kragujevac, 34000 Kragujevac, Serbia; karovic.marko.kg@gmail.com (M.K.); nikola.nedeljkovic@fmn.kg.ac.rs (N.N.); marina.vesovic@fmn.kg.ac.rs (M.V.); andjicmarijana10@gmail.com (M.A.); nevena.lazarevic@fmn.kg.ac.rs (N.L.); sanjadjakovic1996@gmail.com (S.Đ.); 2 Department of Physiology, Faculty of Medical Sciences, University of Kragujevac, 34000 Kragujevac, Serbia; marina.nikolic@fmn.kg.ac.rs (M.N.); drvladakgbg@yahoo.com (V.J.); 3 Center of Excellence for Redox Balance Research in Cardiovascular and Metabolic Disorders, 34000 Kragujevac, Serbia; 4 Department of Human Pathology, 1st Moscow State Medical University IM Sechenov, 119991 Moscow, Russia; 5 Department of Medical Statistics and Informatics, Faculty of Medical Sciences, University of Kragujevac, 34000 Kragujevac, Serbia; jelena.nedeljkovic@fmn.kg.ac.rs; 6 Center for Molecular Medicine and Stem Cell Research, Faculty of Medical Sciences, University of Kragujevac, 34000 Kragujevac, Serbia; 7 Department of Pharmaceutical Chemistry, Faculty of Pharmacy, University of Belgrade, 11221 Belgrade, Serbia; jelena.boskovic@pharmacy.bg.ac.rs (J.B.); vladimir.dobricic@pharmacy.bg.ac.rs (V.D.)

**Keywords:** dexketoprofen, amide derivatives, paw edema, anti-inflammatory activity, antioxidant activity, COX-1, COX-2, molecular docking

## Abstract

In the present study, five novel dexketoprofen amide derivatives with a free carboxyl group in their side chains were synthesized. The in vivo anti-inflammatory potential of dexketoprofen derivatives was evaluated using a carrageenan-induced paw edema model of acute inflammation. Additionally, the local and systemic redox status in rats following acute administration of the compounds was assessed by measuring levels of pro-oxidative markers and the activity of antioxidant enzymes. Among the analyzed molecules, derivatives **2** and **4** exhibited the most potent in vivo anti-inflammatory activity, showing effects comparable to those of the parent compound dexketoprofen. In vitro results revealed that all newly synthesized compounds exhibited low inhibitory activity toward COX-1, whereas only compound **4** showed significant COX-2 inhibition. The stronger binding affinity of derivative **4** for COX-2 in comparison to other tested compounds is likely attributed to its ability to form multiple electrostatic interactions within the enzyme’s active site. Furthermore, compounds **2** and **5** demonstrated efficacy comparable to the parent drug in restoring redox balance, indicating their potential antioxidant properties under acute inflammatory conditions. The findings of this study underscore the therapeutic potential of the novel dexketoprofen amide derivatives as dual-function agents with the capacity to modulate both inflammatory responses and oxidative stress.

## 1. Introduction

Oxidative stress is a pathological condition characterized by an excessive generation of reactive oxygen species (ROS) within cells and tissues, exceeding the capacity of antioxidant defense mechanisms [1,2]. This dysregulation leads to oxidative damage, compromising the structural integrity and function of essential biomolecules [3]. Notably, oxidative stress has been implicated as a key contributor to the pathogenesis of various systemic inflammatory disorders, including rheumatoid arthritis, neurodegenerative diseases, cardiovascular diseases, chronic kidney disease, cancer, and aging [3,4,5]. Moreover, an up-regulation of cyclooxygenase (COX) enzymes, specifically, COX-2, has been observed in chronic arthritis, highlighting the important role of this isozyme in mediating key inflammatory processes that contribute to the tissue damage. Therefore, a comprehensive elucidation of the interplay between ROS and COX-2 is essential for developing novel therapeutic strategies for managing inflammatory conditions [6]. The existing knowledge of ROS involvement in both physiological and pathological processes underscores the need for the development of multifunctional antioxidants capable of modulating oxidative homeostasis under both normal and disease conditions. Within this framework, substantial research efforts have been directed toward the identification, characterization, and therapeutic application of natural antioxidant compounds [7,8,9]. Additionally, significant explorations have been made in the rational design and synthesis of free radical scavengers and antioxidant agents with the potential to attenuate excessive ROS production and enhance endogenous antioxidant defense mechanisms [10,11,12].

The impact of nonsteroidal anti-inflammatory drugs (NSAIDs) on oxidative redox status remains a subject of ongoing scientific debate, as conflicting findings have been reported in the literature. These drugs can exhibit both antioxidant and pro-oxidant properties, depending on various experimental parameters, including the administered dose and the specific agent used to induce oxidative stress [13]. NSAIDs contribute to the generation of ROS through enzymatic pathways involving nicotinamide adenine dinucleotide oxidases, lipoxygenases (LOX), nitric oxide synthases, xanthine oxidoreductases, cytochrome P450 monooxygenases, and COX enzymes. Consequently, oxidative stress is believed to play a crucial role in mediating the adverse effects of NSAIDs, such as gastrointestinal toxicity, nephrotoxicity, and hepatotoxicity [14]. Despite these results, some studies have reported antioxidant properties of NSAID derivatives [15,16,17]. Theodosis-Nobelos and colleagues have demonstrated that amide derivatives of tolfenamic acid exhibit potent antioxidant and anti-inflammatory activity in a model of acute inflammation in Wistar albino rats [18]. The salification of ketoprofen free acid with *L*-lysine has led to the synthesis of ketoprofen lysine salt, which shows improved water solubility and faster dissolution and absorption compared to the parent compound but also exhibits a significant gastroprotective effect [19,20]. In addition, previous in vitro studies on human gastric epithelial cells exposed to ethanol revealed that ketoprofen lysine salt neutralized ethanol- or NSAID-induced cellular injury by inhibiting lipid peroxidation and exerting ROS scavenging effects, largely attributed to the presence of *L*-lysine [21]. These findings suggest that specific NSAID derivatives may exert protective effects against oxidative damage under certain conditions.

Ketoprofen, a member of the propionic acid derivative class of NSAIDs, is widely used in human and veterinary medicine due to its pronounced analgesic, anti-inflammatory, and antipyretic properties [22,23]. The drug is commonly administered in vivo as a racemic mixture of (*R*)- and (*S*)-enantiomers, with only the (*S*)-enantiomer (dexketoprofen) exhibiting COX inhibition and pharmacological activity [24]. Dexketoprofen functions as a potent, non-selective inhibitor of COX-1 and COX-2, while the (*R*)-enantiomer exhibits 100 to 1000 times lower inhibitory capacity [25]. While some authors claimed that interconversion of (*R*)-ketoprofen to the active (*S*)-enantiomer is limited to 10% [26], other studies demonstrated that enantiomeric inversion has been observed in several animal species, except for humans and guinea pigs [27].

**Figure 1:**
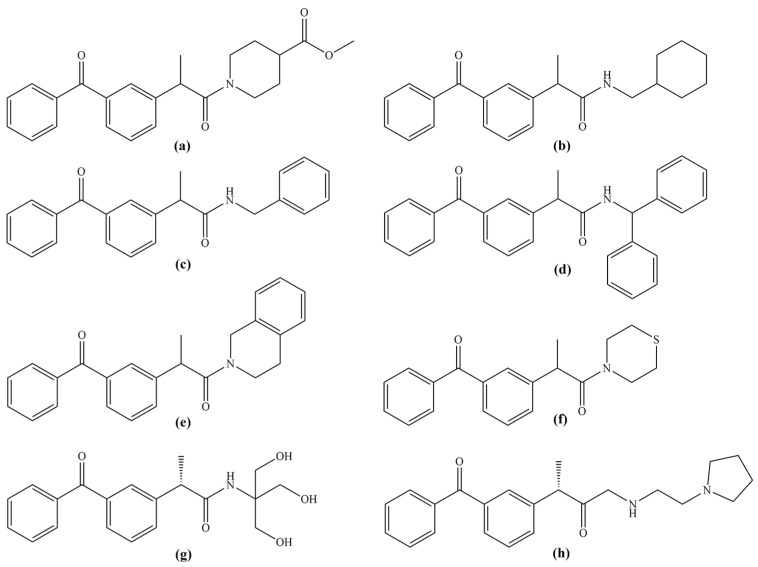
Previously investigated ketoprofen amide derivatives (**a**–**h**) with biological activity.

A variety of NSAID derivatives have been synthesized to minimize side effects, extend the plasma half-life, and enhance the water solubility or lipophilicity. Structural modifications of the NSAIDs’ carboxyl group have been shown to yield bioactive compounds capable of inhibiting both COX and 5-LOX. Additionally, it has been demonstrated that the amidation of NSAIDs can significantly enhance selectivity for COX-2 [28,29,30,31]. Consistent with these findings, various structurally diverse ketoprofen derivatives, including amides [32], esters [33,34], thiadiazoles [35], and aminocarbamates [36], have exhibited a broad spectrum of biological activities in previously published studies. The study conducted by Uludag and colleagues demonstrated that piperidine amide derivatives of ketoprofen exhibit superior anti-inflammatory effects in a mice paw edema model compared to the parent compound [37] (Figure 1a). A previously published study of Rajić and co-workers revealed that various aromatic and cycloalkyl amides of ketoprofen display greater potency as LOX inhibitors than carboxylic acid derivatives, with remarkable efficacy in suppressing lipid peroxidation [38] (Figure 1b–d). Manolov and colleagues synthesized ketoprofen analogs with various N-heterocyclic moieties and found that the 1,2,3,4-tetrahydroisoquinoline derivative showed modest hydrogen peroxide scavenging activity compared to ascorbic acid [39] (Figure 1e). A study by Theodosis-Nobelos and co-workers demonstrated that an amide derivative of ketoprofen featuring a thiomorpholine ring achieved potent inhibition of paw edema, surpassing the anti-inflammatory efficacy of the parent acid [40] (Figure 1f).

To the best of our knowledge, only two studies have focused on the synthesis and biological evaluation of new dexketoprofen derivatives in mice, while no studies have been conducted in rats. Kulabaş and colleagues reported that a polyhydroxylated amide derivative of dexketoprofen demonstrates significant anti-inflammatory activity, accomplishing a 60.9% reduction in mice paw edema (Figure 1g). In vitro assays further revealed that this amide derivative acts as a selective COX-2 inhibitor [41]. Furthermore, a propanamide derivative of dexketoprofen induced a significant drop in paw swelling, with an observed inhibition rate of 40% at 180 min after inflammation induction [42] (Figure 1h).

In this study, we report the synthesis of five novel dexketoprofen amide derivatives. The present research aimed to evaluate their anti-inflammatory effects in vivo using a carrageenan-induced paw edema model and to assess their impact on the local and systemic redox status by measuring pro-oxidant markers and antioxidant enzyme activities in rats. To gain a more comprehensive understanding of the anti-inflammatory properties of dexketoprofen derivatives, in vitro assays were conducted to determine COX-1 and COX-2 enzyme inhibition, while in silico molecular docking studies were performed to investigate the binding model of synthesized compounds into active sites of these isoenzymes.

## 2. Materials and Methods

### 2.1. Chemicals and Instruments

*S*-ketoprofen, glycine methyl ester hydrochloride, carrageenan, *L*-Alanine methyl ester hydrochloride, tetrahydrofuran (THF), *N*-hydroxybenzotriazole (HOBt), *N*,*N*-dicyclohexylcarbodiimide (DCC), and *N*,*N*-dimethylformamide (DMF) were purchased from Sigma-Aldrich (Steinheim, Germany). Methyl 4-aminobutanoate hydrochloride, *L*-phenylalanine methyl ester hydrochloride, *L*-tryptophan methyl ester hydrochloride, and triethylamine (TEA) were obtained from Acros Organics (Geel, Belgium). Chloroform and methanol were bought from JT Baker (Loughborough, UK), while silica gel for preparative thin-layer chromatography (TLC) was purchased from Merck (Darmstadt, Germany). Sodium hydroxide (NaOH) was obtained from Fisher Scientific (Loughborough, UK). Reagents and chemicals that were used for investigation of antioxidative activity were purchased from Sigma Aldrich (St. Louis, MO, USA): sodium tetraborate, sodium chloride, sodium citrate, sulfonylic acid, 5,5′-dithio-bis-(2-nitrobenzoic acid) (DTNB), ethylenediaminetetraacetic acid disodium salt dehydrate, glutathione reduced (GSH), gelatin, trichloroacetic acid (TCA), *tris*(hydroxymethyl)aminomethane, thiobarbituric acid (TBA), *L*-N-(1-naphthyl)-ethylenediamine-dihydrochloride (NEDA), nitro-blue-tetrazolium (NBT), epinephrine, sodium hydroxide, metaphosphoric acid, phosphoric acid, disodium phosphate, ammonium chloride, and sodium carbonate.

The synthesized compounds were structurally characterized through the determination of melting points and spectroscopic techniques, including IR, NMR, HRMS, and MS/MS. Melting points were measured using a Boetius PHMK 05 apparatus (Radebeul, Germany). The ^1^H NMR and ^13^C NMR spectra were recorded on the NMR Bruker Avance III spectrometer (Bruker Biospin GmbH, Rheinstetten, Germany), operating at 400 MHz for ^1^H NMR and 100 MHz for ^13^C NMR. IR spectra were recorded using the ATR-FTIR spectrometer Nicolet iS10 (Thermo Scientific, Madison, WI, USA). Exact masses measurements were performed using the LTQ Orbitrap XL Mass Spectrometer (Thermo Fisher Scientific, Bremen, Germany), while MS/MS analyses were conducted using the TSQ Quantum Access MAX triple quadrupole mass spectrometer (Thermo Fisher Scientific, San Jose, CA, USA), equipped with a heated electrospray ionization source (HESI).

### 2.2. Synthetic Procedures

**Scheme 1:**
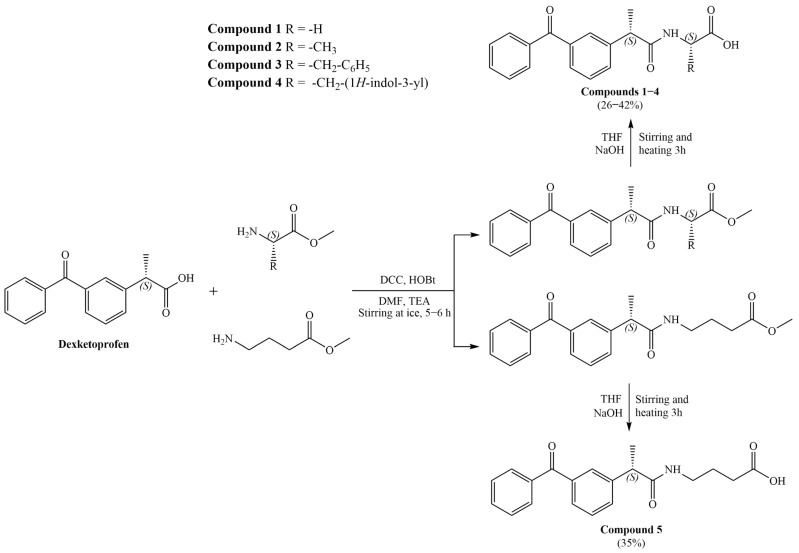
Synthesis of investigated dexketoprofen derivatives.

A series of dexketoprofen amide derivatives (**1**–**5**) was synthesized according to the procedure illustrated in Scheme 1. *S*-ketoprofen (50 mg, 0.2 mmol) was dissolved in *N*,*N*-dimethylformamide (DMF, 4 mL), and this solution was cooled at 0 °C. Subsequently, dicyclohexylcarbodiimide (DCC, 82 mg, 0.4 mmol) and 1-hydroxybenzotriazole (HOBt, 40.5 mg, 0.3 mmol) were introduced into the reaction mixture. The mixture was stirred in an ice bath at the same temperature for 1 h and then left overnight at a temperature not exceeding 8 °C. The corresponding methyl ester of amino acid hydrochloride (0.2 mmol; methyl ester of *L*-glycine, methyl ester of *L*-alanine, methyl ester of *L*-phenylalanine, methyl ester of *L*-tryptophan, methyl ester of 4-aminobutanoic acid) was dissolved in DMF (2 mL), followed by the addition of triethylamine (TEA, 55 μL, 0.4 mmol). Thereafter, the resulting solution was cooled to 0 °C. The mixture of *S*-ketoprofen, DCC, and HOBt was filtered and added dropwise into a previously prepared amino acid ester solution. The reaction was maintained at the magnetic stirrer at 0 °C for 5–6 h, after which the mixture was left to evaporate in the digester at room temperature overnight. The reaction mixtures were purified using preparative TLC using chloroform/methanol 95:5, *v*/*v* as a mobile phase. The resulting amide esters of *S*-ketoprofen were dissolved in tetrahydrofuran (THF, 10 mL) and left on a magnetic stirrer. After that, hydrolysis was carried out by the dropwise addition of 0.1 M NaOH solution at a molar ratio of ester: NaOH = 1:25. The reaction mixture was stirred continuously and heated for 3 h until complete evaporation. Subsequently, the reaction mixtures were dissolved in a mixture of methanol/chloroform = 50:50% *v*/*v* and purified via preparative TLC using chloroform/methanol 70:30, *v*/*v* as a mobile phase.

(*S*)-(2-(3-benzoylphenyl)propanoyl)glycine (**1**)

Yield: 39%. White crystalline solid. Melting point: 209.6–212.5 °C. IR (ATR) ν_max_ (cm^−1^): 1445.87, 1576.38, 1644.78, 2269.20, and 3354.56. **^1^H NMR (400 MHz, DMSO-d_6_) δ ppm** 1.33 (3H, d, J = 7.2 Hz, C**H_3_**_Dexk_), 3.52 (2H, d, J = 4.4 Hz, C**H_2_**), 3.87 (1H, q, J = 6.8 Hz, C**H**_Dexk_), 7.45–7.73 (9H, m, Ar**H**) (Figure S1a). **^13^C NMR (100 MHz, DMSO-d_6_) δ ppm** 19.25, 44.18, 44.99, 128.46, 128.89, 129.04, 130.08, 132.33, 133.12, 137.27, 137.51, 143.48, 172.73, 196.24 (Figure S1b). *m*/*z* = 310.0 [M+H]^+^, 266.06, 223.04, 209.02, 160.04, 100.08, and 74.24. MS [M+Na]^+^ calculated for C_18_H_17_NO_4_ = 334.10498; observed = 334.10399.

((*S*)-2-(3-benzoylphenyl)propanoyl)-*L*-alanine (**2**)

Yield: 31%. White crystalline solid. Melting point: 158–160 °C. IR (ATR) ν_max_ (cm^−1^): 1446.72, 1574.52, 1645.60, 2928.34, and 3314.85. **^1^H NMR (400 MHz, DMSO-d_6_) δ ppm** 1.19 (3H, d, J = 6.8 Hz, C**H_3_**_Dexk_), 1.33 (3H, d, J = 6.8 Hz, C**H_3_**), 3.85 (1H, q, J = 6.2 Hz, C**H**), 7.45–7.72 (9H, m, Ar**H**) (Figure S2a). **^13^C NMR (100 MHz, DMSO-d_6_) δ ppm** 19.45, 19.68, 45.20, 50.25, 128.40, 128.90, 129.00, 129.06, 130.09, 132.36, 133.10, 137.30, 137.51, 143.53, 172.04, 196.22 (Figure S2b). *m*/*z* = 324.1 [M+H]^+^, 306.15, 114.25, 209.17, 280.07, 237.16, and 88.32. MS [M+Na]^+^ calculated for C_19_H_19_NO_4_ = 348.12060; observed = 348.11920.

((*S*)-2-(3-benzoylphenyl)propanoyl)-*L*-phenylalanine (**3**)

Yield: 40%. White powder. Melting point: 193–196 *°*C. IR (ATR) ν_max_ (cm^−1^): 1575.19, 1596.80, 1644.03, 2248.80, and 3390.88. **^1^H NMR (400 MHz, DMSO-d_6_) δ ppm** 1.22 (3H, d, J = 6.8 Hz, C**H_3_**_Dexk_), 2.89, 3.11 (2H, dq, J = 7.2 Hz, J = 6.0 Hz, C**H_2_**), 3.77 (1H, q, J = 7.2 Hz, C**H**_Dexk_), 4.16 (1H, q, J = 4.8 Hz, C**H**), 6.84–7.59 (14H, m, Ar**H**) (Figure S3a). **^13^C NMR (100 MHz, DMSO-d_6_) δ ppm** 19.47, 39.38, 40.64, 45.23, 126.16, 128.11, 128.79, 129.05, 129.94, 130.08, 132.40, 133.11, 139.48, 143.24 (Figure S3b). *m*/*z* = 400.0 [M+H]^+^, 313.05, 208.84, 163.98, 146.99, 118.05, and 103.07. MS [M+Na]^+^ calculated for C_25_H_23_NO_4_ = 424.15193; observed = 424.15147.

((*S*)-2-(3-benzoylphenyl)propanoyl)-*L*-tryptophan (**4**)

Yield: 49%. Yellow powder. Melting point: 182–183.5 °C. IR (ATR) ν_max_ (cm^−1^): 1446.15, 1575.70, 1644.65, 2137.60, 2929.97, and 3305.50. **^1^H NMR (400 MHz, DMSO-d_6_) δ ppm** 1.21 (3H, d, J = 6.8 Hz, C**H_3_**_Dexk_), 3.00 (2H, q, J = 7.2 Hz, C**H_2_**), 3.74 (1H, q, J = 6.8 Hz, C**H**_Dexk_), 4.21 (1H, q, J = 4.8 Hz, C**H**), 6.76–7.72 (14H, m, Ar**H**),10.55, 10.72 (1H, ds, N**H**_Trp_) (Figure S4a). **^13^C NMR (100 MHz, DMSO-d_6_) δ ppm** 19.13, 28.06, 45.22, 55.33, 111.48, 111.79, 118.33, 119.04, 120.92, 123.74, 128.39, 128.57, 128.79, 128.95, 129.04, 130.09, 132.41, 133.09, 136.35, 137.19, 137.49, 143.40, 172.30, 196.21 (Figure S4b). *m*/*z* = 439.1 [M+H]^+^, 309.97, 265.96, 202.94, 159.01, 142.01, and 116.02. MS [M+Na]^+^ calculated for C_27_H_24_N_2_O_4_ = 463.19283; observed = 463.16196.

(*S*)-4-(2-(3-benzoylphenyl)propanamido)butanoic acid (**5**)

Yield: 35%. White crystalline solid. Melting point: 153.5–156.3 °C. IR (ATR) ν_max_ (cm^−1^): 1446.36, 1574.31, 1643.79, 2932.51, and 3350.10. **^1^H NMR (400 MHz, DMSO-d_6_) δ ppm** 1.33 (3H, d, J = 6.0 Hz, C**H_3_**_Dexk_), 1.99 (2H, quint, J = 7.37 Hz, CH_2_C**H_2_**CH_2_), 2.50 (2H, t, J = 7.37 Hz, CH_2_CH_2_C**H_2_**), 3.00 (2H, q, J = 7.5 Hz, C**H_2_**CH_2_CH_2_), 3.70 (1H, q, J = 6.0 Hz, C**H**_Dexk_), 7.47–7.71 (9H, m, Ar**H**), 8.15 (1H, t, N**H**CO) (Figure S5a). **^13^C NMR (100 MHz, DMSO-d_6_) δ ppm** 19.03, 25.87, 34.34, 45.29, 70.24, 128.47, 128.86, 128.93, 129.04, 130.04, 132.08, 133.14, 137.30, 137.51, 143.34, 173.22, 196.23 (Figure S5b). *m*/*z* = 338.0 [M+H]^+^, 320.03, 209.01, 128.05, 102.11, 84.17, and 42.51. MS [M+Na]^+^ calculated for C_20_H_21_NO_4_ = 362.13628; observed = 362.13546.

### 2.3. In Vivo Studies

Healthy male Wistar albino rats (eight weeks old, average body weight of 180–210 g) were obtained from the Military Medical Academy, Belgrade, Serbia, and housed in the vivarium of the Center for Preclinical and Functional Investigations of the Faculty of Medical Sciences, University of Kragujevac, Serbia. The animals were kept under controlled environmental conditions (22 ± 2 °C room temperature, 55 ± 5% humidity, and a 12 h light–dark cycle), with ad libitum access to water and standard rat food. Following a one-week acclimatization period, rats were included in the study to evaluate the anti-inflammatory activity of novel dexketoprofen derivatives. The study protocol was conducted in compliance with the regulations established by the Faculty’s Ethics Committee for the welfare of laboratory animals (protocol code 01-14130, approval date: 29 December 2023) and principles of the Good Laboratory Practice and European Council Directive (86/609/EEC).

#### 2.3.1. Assessment of Acute Toxicity

An acute toxicity study was conducted using healthy male Wistar rats (n = 18), each weighing between 180 and 210 g. The animals were randomly divided into six groups (n = 3 per group). The control group received a single intraperitoneal dose of distilled water containing a few drops of Tween 80. The experimental groups received compounds **1**–**5**, each at a dose of 0.0315 mmol/kg body weight.

Following administration, all animals were housed in single cages and monitored over a 14-day observation period. Body weight and consumption of water and food were recorded daily. Clinical signs of toxicity and behavioral changes were observed and documented during a 2-week period, including assessment of fur and skin condition, eye appearance, respiration, salivation, urination (color), feces consistency, somatomotor activity, and other behavioral patterns. At the end of the observation period, the animals were sacrificed, and the major organs (heart, liver, stomach, and kidney) were excised and weighed [43]. The relative organ weight was calculated using the following formula:

(1) $$\mathrm{Relative}\;\mathrm{organ}\;\mathrm{weight}=\frac{\mathrm{Absolute}\;\mathrm{organ}\;\mathrm{weight}\;\times \;100\%}{\mathrm{Body}\;\mathrm{weight}\;\mathrm{of}\;\mathrm{rat}\;\mathrm{on}\;\mathrm{the}\;\mathrm{day}\;\mathrm{of}\;\mathrm{sacrifice}}$$

#### 2.3.2. Assessment of In Vivo Anti-Inflammatory Potential in Wistar Albino Rats

The in vivo potential of synthesized compounds to exhibit an inhibitory effect on paw swelling was evaluated using a carrageenan-induced rat paw edema model of acute inflammation. Inflammation was induced in all rats by an intraplantar injection of 100 μL of freshly prepared 1% carrageenan solution in distilled water into the left hind paw [44]. A total of 56 male Wistar albino rats were randomly divided into five experimental groups (n = 40 rats, 8 rats per subgroup) and two control groups (n = 16 rats, 8 rats per subgroup).

The experimental groups consisted of rats that received synthesized compounds via intraperitoneal injection immediately following carrageenan administration. The solutions of the investigated molecules were prepared by dissolving the compound in distilled water with the addition of a few drops of Tween 80 [18], whereby each rat received 500 μL of the prepared solution. The control groups included two equal subgroups: the positive control group involved the rats that received an intraperitoneal injection of dexketoprofen solution, prepared in the same way as the test compound solutions, immediately after carrageenan administration. The negative control group included rats that received an intraperitoneal injection of the solvent solution (distilled water with a few drops of Tween 80) at the same time point. The dexketoprofen dose was selected based on the previously published research [45], where a dose of 8 mg/kg in rats is considered equivalent to the therapeutic dose used in humans. The synthesized compounds were administered via intraperitoneal injection at equimolar doses corresponding to the dexketoprofen dose (0.0315 mmol/kg). The control and experimental groups were organized as follows:

- Solvent—negative control group of animals treated with distilled water with a few drops of Tween 80;
- Dexketoprofen—positive control group of animals treated with dexketoprofen;
- Group 1—animals treated with derivative **1**;
- Group 2—animals treated with derivative **2**;
- Group 3—animals treated with derivative **3**;
- Group 4—animals treated with derivative **4**;
- Group 5—animals treated with derivative **5**.

To quantify the anti-edematous effect, the thickness of the left paw tissue of each rat was measured at specific time intervals: immediately before inflammation induction (moment 0) and at 1, 2, 3, and 4 h post-induction (moments 1, 2, 3, and 4, respectively). Tissue thickness was assessed in the middle of the rat paw using a digital vernier caliper (Aerospace, Beijing, China). The percentage inhibition of the paw edema was calculated using the following formula:

% Inhibition = 100 × [1 − (Yt/Yc)](2)

where Yt = average increase in paw thickness in the treated group of rats between two measurement moments, and Yc = average increase in paw thickness in the untreated group of rats between two measurement moments

#### 2.3.3. Evaluation of Synthesized Compounds’ Effect on Systemic and Local Redox State

Following 24 h of carrageenan-induced paw edema inflammation model and intraperitoneal anesthesia induced by ketamine/xylazine (100/10 mg/kg of BW), the animals were sacrificed, while whole-blood samples and edema-affected paw tissues were isolated. After centrifugation of whole blood, plasma and erythrocyte lysate samples were separated for further examination of the applied compounds’ effects on the systemic redox balance state. Pro-oxidative markers including superoxide anion radical (O_2_∙^−^), hydrogen peroxide (H_2_O_2_) nitrites (NO_2_^−^), and index of lipid peroxidation measured as thiobarbituric acid (TBA) reactive substances (TBARS) were determined from plasma samples, while the antioxidant capacity, including superoxide dismutase (SOD) and catalase (CAT) activity, as well as the reduced glutathione (GSH) level were examined in erythrocyte lysate samples [46]. Moreover, in order to examine the effect of the acute administration of novel dexketoprofen derivatives on the local redox state, rats paw tissues were homogenized, and supernatant samples were isolated for further biochemical analysis. Edema-affected paw tissue samples were weighed (100 mg) and homogenized (OmniPrep™ multi-sample homogenizer, Omni International, Kennesaw, GA, USA) in 900 µL phosphate buffer on ice (pH = 7.4), and centrifuged at 12,000 rpm for 20 min. These supernatant samples were used for the determination of the aforementioned pro-oxidative and antioxidative parameters levels. All oxidative stress analyses were performed spectrophotometrically using a Shimadzu UV 1800 spectrophotometer (Kyoto, Japan) [47].

#### Determination of Pro-Oxidative Parameters

Determination of the O_2_∙^−^ level was conducted by adding 950 µL of a Nitro Blue Tetrazolium (NBT) mixture with TRIS buffer in 50 µL of sample, measured at the 550 nm wavelength. The level of H_2_O_2_ was determined during the oxidation of phenol red by H_2_O_2_, which was catalyzed with horseradish peroxidase and measured at the 610 nm wavelength. Indirect measurement of nitrites was performed by the precipitation of samples with 30% sulfo-salicylic acid, followed by vortexing and centrifugation at 3000 rpm for 15 min. The equal volumes of isolated supernatant and Griess’ reagent (containing 1% sulfanilamide in 5% phosphoric acid/0.1% naphthalene ethylenediamine-dihydrochloride) were incubated for 10 min and measured at the 550 nm wavelength. The TBARS concentration was determined by adding 400 µL of sample to 200 µL of TBA in 0.05 M NaOH, followed by 15 min incubation at 100 °C. Measurement was performed at the 530 nm wavelength [48].

#### Determination of Antioxidative Parameters

The epinephrine method was used for SOD determination. By adding 100 µL of sample, the same volume of epinephrine, and 1000 µL of carbonate buffer, the measurement was performed at the 470 nm wavelength. The determination of CAT activity was based on the preparation of a mixture containing 100 µL of sample, 50 µL of catalase buffer, and 1000 µL of 10 mM H_2_O_2_, which was measured at the 230 nm wavelength. The level of GSH was measured at the 412 nm wavelength, and the reaction was based on GSH oxidation by 5,5′-dithiobis-(2-nitrobenzoic acid; DTNB), which resulted in GSSG and 5-thio-2-nitrobenzoic acid (TNB) formation [48].

### 2.4. In Vitro Investigation of COX-1 and COX-2 Inhibitory Activity

The inhibitory activity of the tested compounds against COX-1 and COX-2 isoforms was assessed using fluorometric cyclooxygenase inhibitor screening kits (Catalog Numbers ab204698 and ab283401, respectively; Abcam, Cambridge, UK). These assays detect the formation of prostaglandin G2, a product of COX enzymatic activity, via fluorometric measurement.

Stock solutions of the test compounds were prepared at a concentration of 5 mM in DMSO and subsequently diluted with the same solvent to obtain the test solutions. For the assay preparation, 2 μL of each test solution was diluted with 8 μL of COX assay buffer. The solvent control (SC) consisted of 2 μL of DMSO and 8 μL of assay buffer, while the enzyme control (EC) contained 10 μL of assay buffer alone. Sample wells (S) received 10 μL of the diluted test solutions. All other steps were in accordance with the manufacturer’s protocol and the previously published article [49]. Fluorescence was measured kinetically over a 10 min period at 25 °C, using the excitation and emission wavelengths of 535 nm and 587 nm, respectively. The percentage of inhibition was calculated as follows:

(3) $$\%\;\mathrm{Relative}\;\mathrm{Inhibition}=\frac{\mathrm{Slope}\;\mathrm{of}\;\mathrm{EC}-\mathrm{Slope}\;\mathrm{of}\;S}{\mathrm{Slope}\;\mathrm{of}\;\mathrm{EC}}\times 100$$

Fluorescence measurements were conducted on a BioTek Synergy LX multi-mode reader (Winooski, VT, USA).

### 2.5. In Silico Molecular Docking Studies

The binding affinities of dexketoprofen and its amide derivatives toward the COX-1 and COX-2 isozymes were evaluated through molecular docking studies using AutoDock Vina version 1.1.2 [50]. Prior to docking, energy minimization of the ligand conformers was conducted by Chem3D Ultra 7.0 [51], applying the AM1 semiempirical method. The crystal structures of COX-1 (PDB ID: 3N8Z) [52] and COX-2 (PDB ID: 3PGH) [53] were retrieved from the RCSB Protein Data Bank. Pre-processing of the target proteins involved the removal of non-essential chains, co-crystallized ligands, water molecules, and cofactors, and was carried out using BIOVIA Discovery Studio [54]. Final preparation of the protein structures was performed in AutoDock Tools [55], where polar hydrogen atoms were added, and Kollman partial charges were assigned. A flexible ligand-rigid protein docking approach was employed, restricting the docking process to chain A of each enzyme. Binding sites were defined based on the coordinates of the co-crystallized ligand, flurbiprofen. Grid parameters were set with a spacing of 0.375 Å and dimensions of 42 × 22 × 24 grid points. The grid box centers were defined as follows: COX-1 at –20.975, 50.155, and 10.484 and COX-2 at 25.415, 21.973, and 14.891. Binding affinity was assessed in terms of interaction type, number of non-covalent contacts, and docking scores (ΔG). The three-dimensional visualization of ligand–enzyme interactions for the top-ranked docking poses was performed using PyMOL version 2.5.5 [56].

## 3. Results and Discussion

### 3.1. General Procedure for the Synthesis of Amide Derivatives of Dexketoprofen

The synthetic strategy involved the conversion of the carboxyl group of dexketoprofen into the amide moiety in the presence of DCC, HOBt, and corresponding amino acid methyl ester, followed by ester hydrolysis to obtain compounds **1**–**5** with a free carboxyl group in their side chains (Scheme 1).

The synthesis was carried out following an established protocol for structurally related compounds [29,30], using *S*-ketoprofen as a starting compound. This synthetic strategy, involving functionalization of the carboxyl group and extension of the spacer between the carboxyl group and the aromatic moiety of dexketoprofen, was implemented with the aim to enhance its anti-inflammatory and antioxidant activities.

In the ^1^H NMR spectrum of the L-glycine derivative (compound **1**), the CH_3_ group from the dexketoprofen scaffold yielded a doublet at 1.33 ppm. The methylene group of L-glycine yielded a doublet at 3.52 ppm, while the quartet signal of hydrogen at the chiral carbon appeared at 3.87 ppm. Aromatic protons produced signals in the range from 7.45 to 7.73 ppm. In the ^13^C NMR spectrum, dexketoprofen’s methyl group produced a signal at 19.25. Signals of the chiral carbon and methylene group carbon were located at 44.18 and 44.99 ppm, respectively, while signals of the benzene nuclei were between 128.46 and 143.48 ppm. Two carbons from the amide and keto group provided signals at 172.73 and 196.24 ppm, respectively. The NMR spectra of all synthesized compounds are presented in the Supplementary Materials. The HRMS spectrum showed the adduct [M+Na]^+^ with the *m*/*z* ratio 334.10399 (calculated value: 334.10498; error: 2.96 ppm).

### 3.2. Evaluation of Acute Toxicity

**Figure 2:**
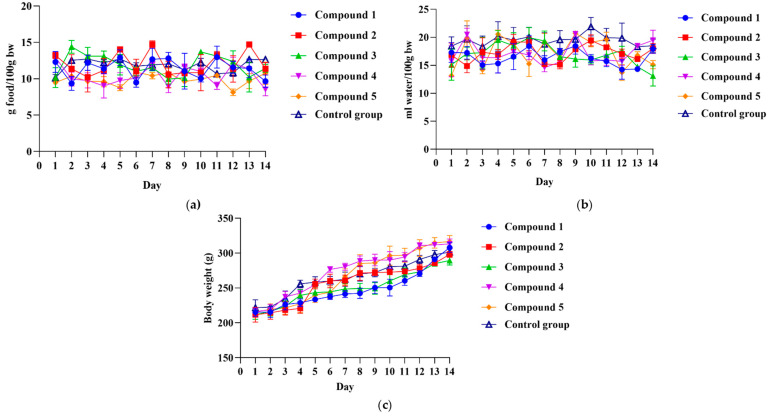
Results of food intake (**a**), water intake (**b**), and body weight (**c**).

The potential toxicity of the newly synthesized derivatives of dexketoprofen on vital organs was evaluated with the aim to assess their safety profile. Throughout the 14-day observation period, no clinical signs of systemic toxicity were detected in rats treated with compounds **1**–**5**. Daily monitoring indicated that the body weight gain, as well as water and food intake, remained comparable between the treated and control groups (Figure 2).

It is important to note that no alterations in general appearance or behavior were observed in treated animals. Monitored parameters, including physical activity, fur and skin condition, mucous membrane appearance, urination color, feces consistency, and aggression, remained within normal ranges, showing no deviations from the control group. In addition, statistical analysis revealed no significant differences in the relative weights of major organs (liver, kidney, stomach, and heart) between treated and untreated animals (Table 1).

### 3.3. In Vivo Evaluation of the Anti-Inflammatory Potential

The in vivo anti-inflammatory activity of the synthesized compounds was evaluated using the carrageenan-induced paw edema model, a well-established and widely used method for assessing the efficacy of potential anti-inflammatory agents in acute inflammation.

**Figure 3:**
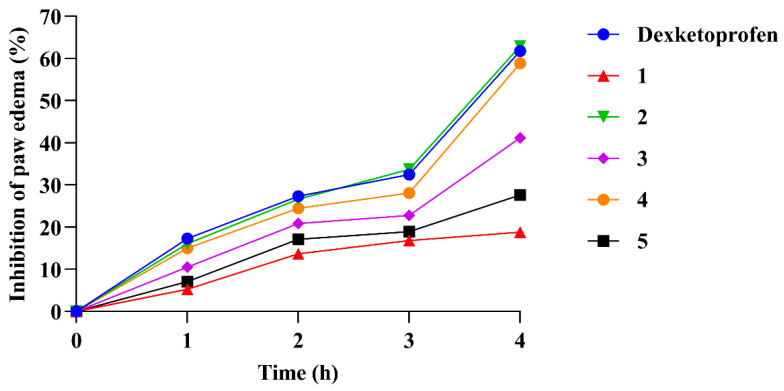
Percentage of paw edema inhibition in rats treated with compound **1** (dexketoprofen glycine derivative), compound **2** (dexketoprofen alanine derivative), compound **3** (dexketoprofen phenylalanine derivative), compound **4** (dexketoprofen tryptophan derivative), compound **5** (dexketoprofen GABA derivative), and dexketoprofen.

Most of the tested derivatives have shown certain anti-inflammatory potential. Treatment with compounds **2** and **4** significantly reduced the edema diameter in the 2nd hour post-carrageenan administration compared to the control group (*p* < 0.05), with this effect persisting until the end of the observed period (*p* < 0.01), similar to the standard anti-inflammatory drug dexketoprofen. However, compound **2** can be singled out, showing the highest percentage of inhibition compared to the control (62.941%), closely matching the effect of the standard dexketoprofen (61.765%). In contrast, compound **5** showed slight anti-inflammatory potential only in the 4th hour of observation (27.647%), compound **3** only in the 2nd hour of observation (20.863%), while compound **1** did not achieve an anti-inflammatory effect at any measurement times (*p* > 0.05, Table 2, Figure 3).

It is well known that paw edema formation following carrageenan administration follows a biphasic inflammatory event pattern. Namely, the initial early phase occurs 0–2.5 h post-carrageenan injection and is associated with the release of mediators such as histamine, serotonin, and leukotrienes, as well as increased vascular permeability. The second phase occurs 2.5–6 h after carrageenan injection, and in this phase, COX and LOX pathways become more prominent, leading to the release of leukotrienes, prostaglandins, and inflammatory cytokines, all together leading to edema formation [57,58]. Our study showed that the 1st or 2nd hour represents the peak of edema formation in all groups (Table 2), in that way confirming the development of a local acute inflammatory reaction. Given that compounds **2**–**5** exhibited their strongest anti-inflammatory effects during the later stage of carrageenan-induced inflammation, it can be suggested that the possible mechanism of their anti-inflammatory action may involve modulation of COX enzyme activity. Namely, as expected, the parent compound dexketoprofen proved to be a potent, non-selective inhibitor of both COX-1 and COX-2, with a preference for COX-1 inhibition. In contrast, none of the five tested derivatives demonstrated significant COX-1 inhibition capacity, while only compound **4** exhibited appreciable inhibitory activity against COX-2. This aligns with the fact that compound **4** also demonstrated significant in vivo anti-inflammatory efficacy, suggesting COX inhibition as a potential mechanism of its action. However, compound **2**, which showed the highest percentage of paw edema inhibition, did not exhibit COX inhibitory activity in vitro. Therefore, it is plausible to propose that its mechanism of action may involve other molecular mechanisms of inflammatory cytokine release suppression rather than COX inhibition. Further studies focusing on cytokine profiling will be crucial for clarifying the precise mechanism of action of compound **2**, given that this aspect was beyond the scope of the current research. Tziona et al. have shown that the amide derivative of ketoprofen with a beta-alanine linker exhibited 91% of paw edema inhibition after 3.5 h post-carrageenan usage. These results support our findings on compound **2**, which is an alanine-derivative of dexketoprofen, being the most potent anti-inflammatory agent of the total five tested (62.941%) [59]. Another study that was also focused on the anti-inflammatory activity of amide and ester ketoprofen derivatives showed comparable or even higher anti-inflammatory activity to the parent drug, especially 3 and 4.5 h post-carrageenan injection [37]. Since prostaglandins are secreted in the 3rd and 4th hours after carrageenan injection, it was indicated that all synthesized derivatives still maintained the inhibition of the arachidonic acid pathway effectively. Among the chosen arylpropionic acids, esterification of ketoprofen to its lipophilic derivatives produced more potent compounds in terms of analgesic and anti-inflammatory activity, while the amidation was not as effective. Propanamide derivatives of ketoprofen were proved to inhibit paw edema formation in mice, also in the second phase of inflammation, as did the tested compounds in this research, which exhibited a pronounced anti-edematous effect in rats [42].

### 3.4. Effect of Novel Compounds on Systemic Oxidative Stress Parameters

**Figure 4:**
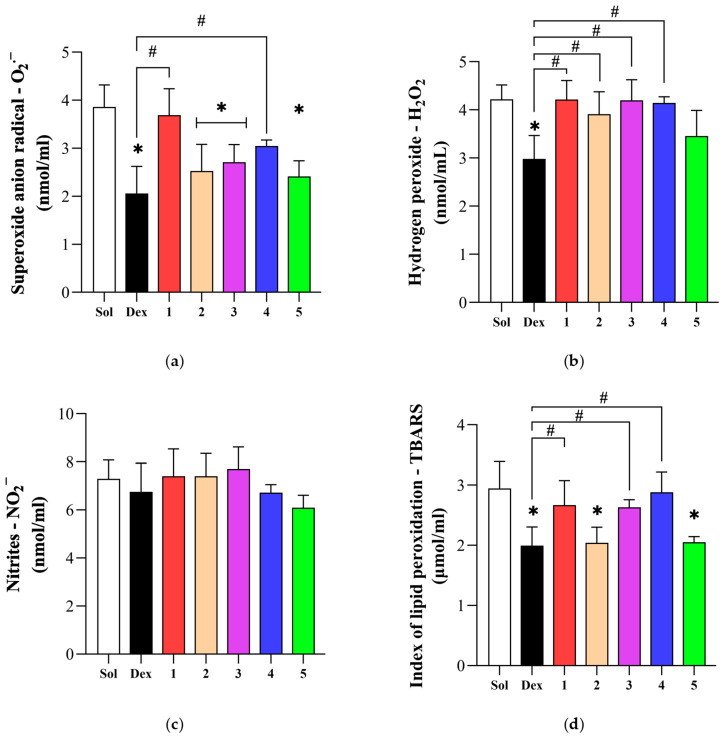
Effect of applied dexketoprofen derivatives on the systemic pro-oxidative parameters. (**a**) Superoxide anion radical—O_2_∙^−^; (**b**) hydrogen peroxide—H_2_O_2_; (**c**) nitrites—NO_2_^−^; (**d**) index of lipid peroxidation—TBARS. Sol—solvent group; Dex—dexketoprofen group; 1—dexketoprofen glycine derivative group; 2—dexketoprofen alanine derivative group; 3—dexketoprofen phenylalanine derivative group; 4—dexketoprofen tryptophan derivative group; 5—dexketoprofen GABA derivative group. Data are presented as means ± standard deviation. Statistical significance at the level *p* < 0.05 * compared to Sol and ^#^ compared to Dex group.

After 24 h of inducing acute inflammation, the O_2_∙^−^ value was significantly lower in the Dex group compared to the untreated rats, as well as in the groups treated with derivatives **2**, **3**, and **5** (*p* < 0.05). Conversely, a significantly higher concentration of this pro-oxidative marker was found in the blood of rats injected with novel dexketoprofen compounds **1** and **4**, comparing to the positive Dex control group (*p* < 0.05). There was no significant difference in the O_2_∙^−^ value between the Dex group and animals treated with the **2**, **3**, and **5** derivatives (*p* > 0.05) (Figure 4a). Administration of dexketoprofen led to a significantly lower H_2_O_2_ level compared to its concentration found in the blood of the rats that received a solvent (*p* < 0.05). However, all the tested compounds significantly increased the value of the H_2_O_2_ compared to the Dex group (*p* < 0.05), except for derivative **5**, whose H_2_O_2_ level did not differ significantly compared to the Dex rats (*p* > 0.05) (Figure 4b). While changes in the nitrites value did not vary significantly between groups (*p* > 0.05) (Figure 4c), we obtained interesting results in the TBARS systemic level. Namely, dexketoprofen injection significantly decreased the TBARS concentration compared to the Sol group, as well as rats treated with compounds **1**, **3**, and **4** (*p* < 0.05). It is noteworthy to emphasize that the novel derivatives **1** and **5** succeeded in significantly reducing the TBARS level compared to untreated rats (*p* < 0.05), with no significant difference when compared to the Dex group (*p* > 0.05) (Figure 4d).

**Figure 5:**
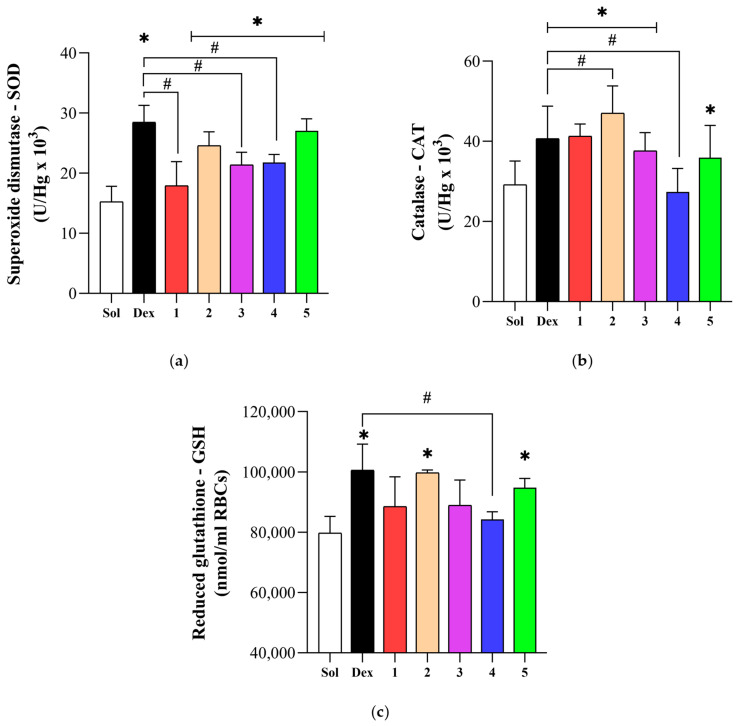
Effect of applied dexketoprofen derivatives on the systemic antioxidative parameters. (**a**) Superoxide dismutase—SOD; (**b**) catalase—CAT; (**c**) reduced glutathione—GSH. Sol—solvent group; Dex—dexketoprofen group; 1—dexketoprofen glycine derivative group; 2—dexketoprofen alanine derivative group; 3—dexketoprofen phenylalanine derivative group; 4—dexketoprofen tryptophan derivative group; 5—dexketoprofen GABA derivative group. Data are presented as means ± standard deviation. Statistical significance at the level *p* < 0.05 * compared to Sol and ^#^ compared to Dex group.

Carrageenan-induced acute inflammation markedly disturbed antioxidant capacity due to the significantly reduced activity of SOD and CAT, as well as the GSH level, in untreated rats compared to Dex (*p* < 0.05) (Figure 5a–c). Also, the SOD activity was improved in rats injected with novel dexketoprofen compounds **2**–**5** compared to Sol (*p* < 0.05), but significantly lower activity of this enzyme was found after treatment with compounds **1**, **3**, and **4** related to Dex rats. Among all tested derivatives, compounds **2** and **5** induced the most prominent SOD activity increase, with no statistical differences compared to the Dex group (*p* > 0.05) (Figure 5a). The same trend was observed in CAT activity, but compound **2** stood out as the derivative with the highest potential to increase CAT activity compared to Dex, as well as to the other examined compounds (*p* < 0.05). Only derivative **4** decreased this enzyme activity relative to the positive control group, reaching the value like the untreated group of rats (Figure 5b). The greatest antioxidant potential of compounds **2** and **5** among the other tested derivatives was also proved through the significantly increased GSH level compared to the untreated rats (*p* < 0.05), while the other dexketoprofen derivatives did not induce any changes in the GSH value (*p* > 0.05). Compared to the Dex group, only compound **4** significantly reduced the GSH concentration (*p* < 0.05) (Figure 5c).

### 3.5. Effect of Novel Compounds on Local Oxidative Stress Parameters

**Figure 6:**
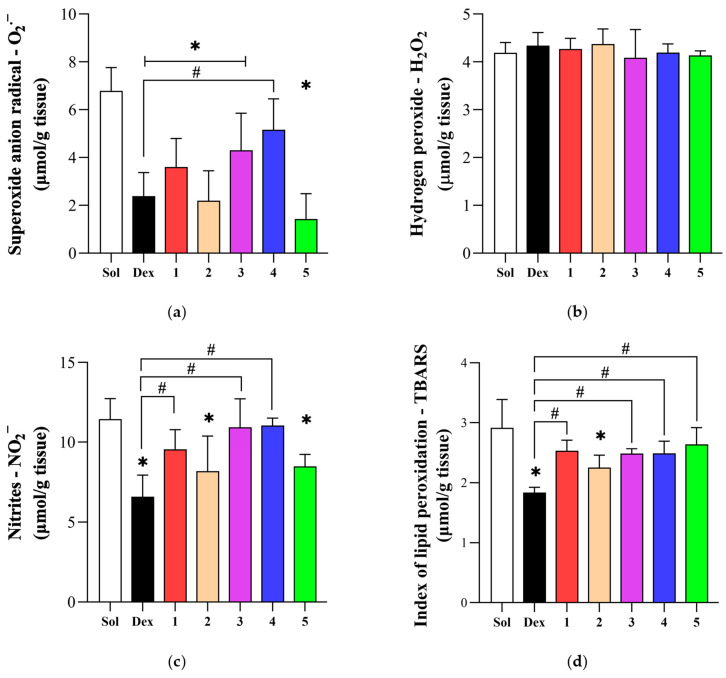
Effect of applied dexketoprofen derivatives on the local pro-oxidative parameters. (**a**) Superoxide anion radical—O_2_∙^−^; (**b**) hydrogen peroxide—H_2_O_2_; (**c**) nitrites—NO_2_^−^; (**d**) index of lipid peroxidation—TBARS. Sol—solvent group; Dex—dexketoprofen group; 1—dexketoprofen glycine derivative group; 2—dexketoprofen alanine derivative group; 3—dexketoprofen phenylalanine derivative group; 4—dexketoprofen tryptophan derivative group; 5—dexketoprofen GABA derivative group. Data are presented as means ± standard deviation. Statistical significance at the level *p* < 0.05 * compared to Sol and ^#^ compared to Dex group.

Except for derivative **4**, the potential of all tested compounds, as well as dexketoprofen, to improve the local redox status was reflected through the significantly decreased O_2_∙^−^ concentration in inflamed tissue compared to untreated rats (*p* < 0.05) (Figure 6a). While no statistically significant differences were found in the H_2_O_2_ value (*p* > 0.05) (Figure 6b), the nitrites level was markedly reduced in Dex rats and animals treated with compounds **2** and **5** (*p* < 0.05). Conversely, the other dexketoprofen derivatives increased the nitrite levels in rats’ paw tissue compared to the positive control group (*p* < 0.05) (Figure 6c). Compound **2** stood out as the only derivative that was able to reduce the TBARS level compared to Sol rats (*p* < 0.05), with no statistical differences compared to the Dex group (*p* > 0.05). Moreover, all other compounds significantly increased the TBARS concentration in inflamed rats paw tissue in comparison to the positive control group of rats (*p* < 0.05) (Figure 6d).

**Figure 7:**
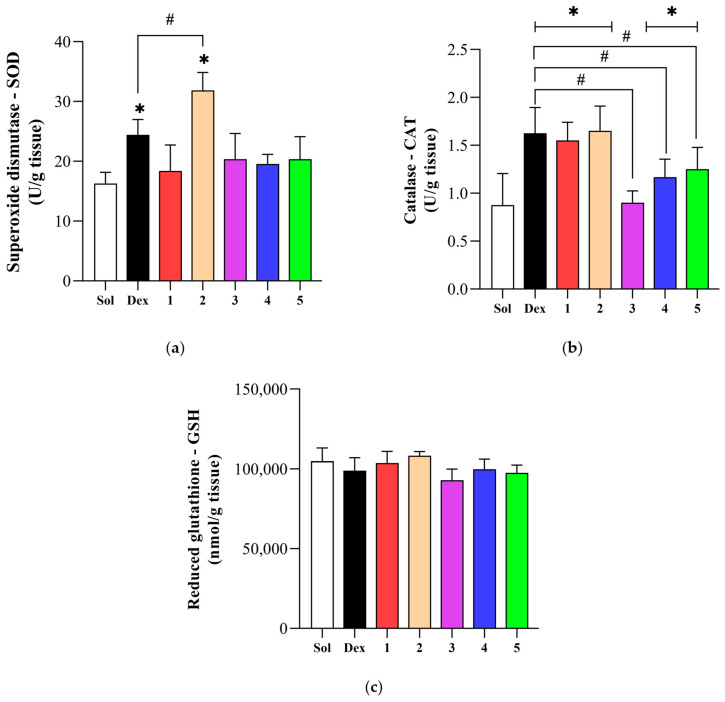
Effect of applied dexketoprofen derivatives on the local antioxidative parameters. (**a**) Superoxide dismutase—SOD; (**b**) catalase—CAT; (**c**) reduced glutathione—GSH. Sol—solvent group; Dex—dexketoprofen group; 1—dexketoprofen glycine derivative group; 2—dexketoprofen alanine derivative group; 3—dexketoprofen phenylalanine derivative group; 4—dexketoprofen tryptophan derivative group; 5—dexketoprofen GABA derivative group. Data are presented as means ± standard deviation. Statistical significance at the level *p* < 0.05 * compared to Sol and ^#^ compared to Dex group.

The activity of SOD was remarkably improved with dexketoprofen compared to the untreated rats. However, among all tested compounds, only derivative **2** was able to increase the SOD activity compared to the negative control group, but also to Dex rats (*p* < 0.05) (Figure 7a). Except for derivative **3** (*p* > 0.05), the CAT activity was improved in all groups compared to untreated rats (*p* < 0.05). In addition, compounds **3**–**5** reduced CAT activity compared to the other two tested derivatives, as well as to the Dex group (*p* < 0.05) (Figure 7b). On the other hand, there were no prominent changes in the GSH level in inflamed tissue between the examined groups of rats (*p* > 0.05) (Figure 7c).

In the first part of the in vivo investigation, we confirmed that dexketoprofen and its derivative **4** exert an anti-inflammatory effect by inhibiting COX enzymes, thereby reducing the synthesis of pro-inflammatory prostaglandins. However, increasing evidence suggests that the pharmacological profile of NSAIDs also includes modulation of oxidative stress pathways. It has been shown that NSAIDs can affect both the generation of ROS and the activity of endogenous antioxidant systems, which can significantly impact the course of inflammation and tissue injury [60,61].

Therefore, our investigation was designed to evaluate the effects of novel dexketoprofen derivatives both on the systemic and local redox status in a rat model of carrageenan-induced acute inflammation. As expected, the acute inflammation induced by a single carrageenan injection significantly deranged the oxidative balance, as evidenced by elevated levels of pro-oxidative markers and impaired antioxidant defense, both in blood and animals’ paw samples. These findings are consistent with previous investigations demonstrating that acute inflammation disrupts redox homeostasis and antioxidant defenses [62,63]. Treatment with dexketoprofen effectively alleviated oxidative stress both systemically and locally, demonstrating its high antioxidative potential in inflammatory conditions. Moreover, the novel dexketoprofen compounds, particularly **2** and **5**, showed comparable modulation of the redox balance in protecting cells from oxidative stress compared to the parent drug. These effects are thought to contribute to their overall anti-inflammatory efficacy and may also play a role in minimizing tissue damage during acute and chronic inflammation [64].

The findings from our study are in line with previous studies investigating the antioxidative effects of dexketoprofen in different models. According to the literature data, dexketoprofen administration reduced plasma malondialdehyde (MDA) levels and enhanced antioxidant enzymes activity, such as the CAT, SOD, and glutathione peroxidase (GPx) levels, in a rat renal ischemia-reperfusion injury model [65]. Additionally, dexketoprofen pretreatment decreased MDA levels and neutrophil infiltration in a rat liver ischemia-reperfusion injury model, suggesting its protective role against oxidative damage [66].

On the other hand, some studies have reported that dexketoprofen may exacerbate tissue injury under certain conditions. Koksal and colleagues observed that dexketoprofen administration aggravated renal injury in rats subjected to ischemia-reperfusion, as evidenced by elevated MDA, increased histological damage scores, and higher blood urea nitrogen (BUN) and creatinine levels. These adverse outcomes suggest that under certain pathological conditions or dosing regimens, dexketoprofen may exacerbate oxidative tissue injury rather than ameliorate it. Such discrepancies across studies may be attributed to several variables, including differences in animal models, dosing protocols, timing and route of administration, and tissue-specific susceptibilities [65].

Furthermore, our results regarding the antioxidative activity of novel dexketoprofen derivatives, specifically, compounds **2** and **5**, support the concept that structural modifications of NSAIDs can modulate their redox-related properties. These derivatives demonstrated even comparable efficacy in restoring the redox balance compared to dexketoprofen, suggesting that their chemical alterations may optimize interactions with oxidative signaling pathways or enhance bioavailability at inflamed sites. This aligns with earlier studies indicating that amidation of NSAIDs can yield derivatives with improved pharmacological and safety profiles [67].

Taken together, these findings underscore the therapeutic potential of dexketoprofen derivatives not only as anti-inflammatory agents but also as modulators of oxidative stress, which is increasingly recognized as a central component of inflammatory tissue damage. The dual mechanism of action, inflammation-suppressing effect and redox modulation, may offer a broader spectrum of efficacy, particularly in conditions where oxidative imbalance plays a critical role.

### 3.6. Investigation of COX-1 and COX-2 Inhibitory Properties

The inhibitory activity of the tested compounds against COX-1 and COX-2 enzymes was evaluated to elucidate their potential anti-inflammatory mechanisms. Enzyme inhibition was assessed using commercial fluorometric assay kits. The results of the COX inhibition assays are summarized in Table 3 and are expressed as mean half-maximal inhibitory concentration (IC_50_) values ± standard error, based on three independent measurements.

None of the synthesized compounds exhibited more than 50% inhibition of COX-1 at concentrations below 100 μM, indicating weak COX-1 inhibitory activity. In contrast, compound **4** demonstrated measurable COX-2 inhibition, with an IC_50_ value that could be determined (37.55 μM). These findings suggest that the anti-inflammatory effect observed for compound **4** is likely mediated through selective inhibition of COX-2 rather than COX-1. The notably higher COX-2 inhibitory activity of compound **4**, compared to the other derivatives, suggests that the presence of a tryptophan residue in the side chain, as an aromatic amino acid, significantly contributes to the observed effect. Furthermore, the observed COX-2 inhibition, along with the most pronounced reduction in inflammation in the rat paw edema model, positions compound **4** as a promising starting point for further development as an anti-inflammatory agent.

The results presented in Table 3 confirm that dexketoprofen is a more potent inhibitor of COX-1 than COX-2, as obtained in previously published studies [41,59]. In comparison to dexketoprofen derivatives, the reference drug exhibited superior inhibitory activity against both COX-1 and COX-2, indicating that the chemical modifications applied to the newly synthesized compounds did not enhance their COX inhibitory profiles. The observed discrepancy, characterized by the potent in vivo anti-inflammatory activity and generally low in vitro inhibition capacity of COX isozymes, suggests that dexketoprofen amide derivatives may exert their anti-edematous effects via a molecular mechanism distinct from COX inhibition. Alternatively, these compounds might function as prodrugs, undergoing enzymatic activation by amidases in vivo to release free dexketoprofen. Accordingly, future research should explore alternative mechanisms that may underlie the anti-inflammatory activity of these compounds.

### 3.7. Molecular Docking Simulation Studies

Molecular docking studies were conducted to investigate the interactions of the tested compounds with the active sites of COX-1 and COX-2 enzymes after in vitro enzyme inhibition tests. Dexketoprofen, a well-known NSAID, was used as a reference drug. The core structure of COX-1 and COX-2 enzymes is conserved across various species, with their amino acid sequences showing approximately 60–65% similarity among mammalian species. Although the active sites of both COX isozymes exhibit considerable structural resemblance, particularly in the presence of residues Arg120, Tyr355, and Glu524, which contribute to a partial constriction at the entrance of the active site, the site ultimately opens into a narrow and elongated hydrophobic channel that extends toward the catalytic binding region [41]. Despite these similarities, a key structural difference arises from the substitution of isoleucine at position 523 in COX-1 with a less bulky valine residue in COX-2. The hydrophobic portion of the active site in both COX isoforms is composed of 24 amino acid residues. In addition to this variation, COX-2 features an extra hydrophilic side pocket, positioned as an extension of the main active site cavity. The formation of this pocket results from the replacement of Ile434 and His513 in COX-1 with the smaller Val434 and the more basic Arg513 in COX-2. These substitutions not only increase the size of the pocket (approximately a 20% increase in the binding pocket volume of COX-2) but also alter its chemical environment, allowing for additional interactions with certain inhibitors. This structural adaptation is thought to facilitate the selective binding of COX-2 inhibitors, such as celecoxib, by providing unique binding opportunities that are not present in COX-1 [68,69].

**Figure 8:**
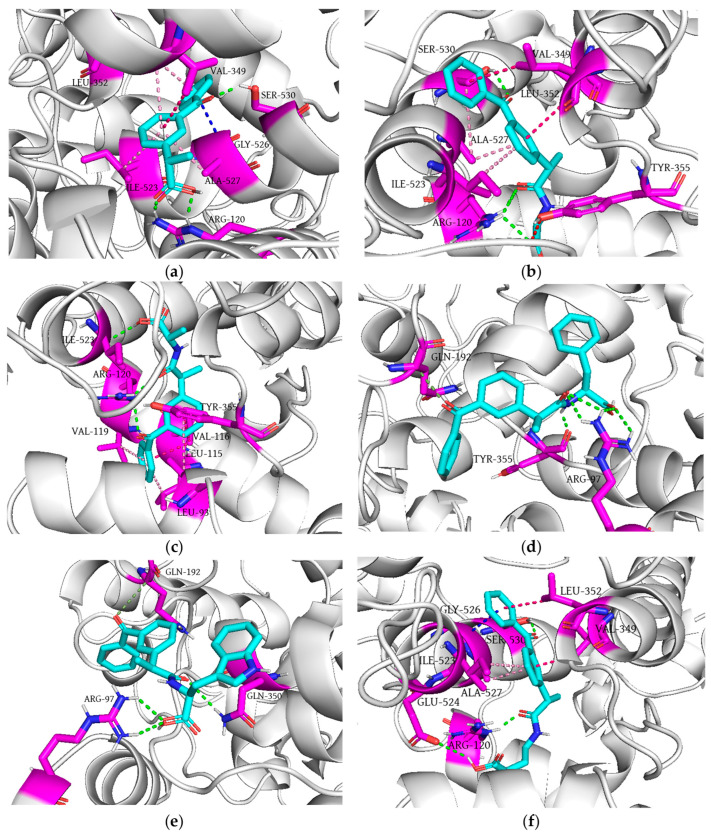
Three-dimensional representation of dexketoprofen (**a**), compound **1** (**b**), compound **2** (**c**), compound **3** (**d**), compound **4** (**e**), and compound **5** (**f**) binding interactions within the binding pocket of COX-1. Illustrated interactions include conventional hydrogen bonds (green dashed lines), C–H hydrogen bonds (smudge dashed lines), π–σ interactions (hot-pink dashed lines), π–π interactions (magenta dashed lines), π–alkyl interactions (pink dashed lines), amide–π interactions (blue dashed lines), and unfavorable steric bumps (red dashed lines).

Molecular docking results demonstrated that dexketoprofen forms the most stable complex with the COX-1 enzyme, yielding a docking score of −8.9 kcal/mol (Table 4). The optimal positioning of the control compound within the enzyme’s active site was stabilized by three conventional hydrogen bonds. Specifically, the carboxyl group of dexketoprofen formed two hydrogen bonds with the guanidinium group of Arg120 (hydrogen bond donor), while the carbonyl oxygen of the keto group, functioning as a hydrogen bond acceptor, established a third conventional hydrogen bond with the hydroxyl group of Ser530. Additional stabilization was provided by hydrophobic interactions between the benzene rings of dexketoprofen and hydrophobic residues Val349, Leu352, Ile523, Gly526, and Ala527 (Figure 8a).

Among the newly synthesized compounds, the aromatic amino acid derivatives (compounds **3** and **4**) exhibited the most favorable binding affinities, both achieving docking scores of −8.8 kcal/mol (Table 4). Due to likely increased steric bulk, these compounds were positioned near the entrance of the COX-1 binding site. This region favored the formation of multiple conventional hydrogen bonds involving the carboxyl group of compound **4**, or both the carboxyl and amide groups of compound **3**, with the guanidinium moiety of Arg97. A common feature observed in both compounds was the presence of a carbon–hydrogen bond formed between the carbonyl oxygen of the keto group (hydrogen bond acceptor) and the side chain of Gln192. The primary distinction in their binding modes was observed in the additional hydrogen bonding: compound **3** formed a conventional hydrogen bond through its -NH group with the carbonyl oxygen of Tyr355, whereas compound **4** engaged in a hydrogen bond via its amide oxygen with the amide group of Gln350 (hydrogen bond donor) (Figure 8).

The remaining synthesized compounds exhibited higher docking scores, ranging from −8.5 to −7.6 kcal/mol, despite forming a greater number of binding interactions (Table 4). This suggests that these interactions were insufficient to confer significant COX-1 binding affinity. Furthermore, the increased steric bulk of these derivatives likely impaired optimal accommodation within the enzyme’s active site, thereby reducing their inhibitory potential.

**Figure 9:**
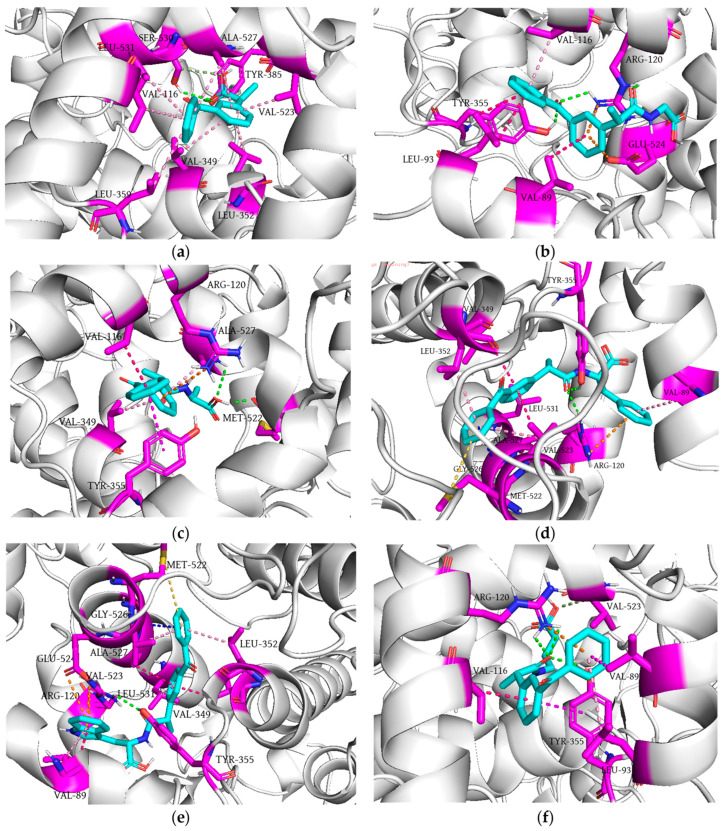
Three-dimensional representation of dexketoprofen (**a**), compound **1** (**b**), compound **2** (**c**), compound **3** (**d**), compound **4** (**e**), and compound **5** (**f**) binding interactions within the binding pocket of COX-2. Illustrated interactions include conventional hydrogen bonds (green dashed lines), C–H hydrogen bonds (smudge dashed lines), π–σ interactions (hot-pink dashed lines), π–π interactions (magenta dashed lines), π–alkyl interactions (pink dashed lines), amide–π interactions (blue dashed lines), and unfavorable steric bumps (red dashed lines).

The interaction of dexketoprofen and COX-2 is characterized by the formation of two conventional hydrogen bonds. The carbonyl oxygen atom of the ligand’s carboxyl group (hydrogen bond acceptor) interacted with phenolic and hydroxyl groups of residues Tyr385 and Ser530, respectively (hydrogen bond donors). In addition, the methylene group of residue Ser530, as a hydrogen donor, formed a carbon–hydrogen bond with the hydroxyl oxygen of dexketoprofen’s carboxyl group. The ligand–protein complex is further stabilized through a grid of multiple π–alkyl interactions that benzene rings establish with residues Val116, Val349, Leu352, Leu359, Val523, Ala527, and Leu531. Molecular docking analysis revealed that all newly synthesized compounds (except for derivative **2**) form conventional hydrogen bonds via the carbonyl oxygen atom of their amide group, acting as a hydrogen bond acceptor, with the guanidinium group of Arg120 and the phenolic group of Tyr355. In contrast, derivative **2** engaged in hydrogen bonding through the hydroxyl moiety of its carboxyl group, interacting with the carbonyl oxygen of Met522 and the amino group of Ala527 (Figure 9). Docking score values indicated that dexketoprofen forms the least stable complex, highlighting the importance of these conventional hydrogen bonds in stabilizing interactions with the COX-2 binding site. In contrast to its amide derivatives, dexketoprofen did not form any electrostatic interactions, which may contribute to its comparatively weaker binding affinity for COX-2. Among the newly synthesized derivatives, compound **4** exhibited the most favorable docking score (−10.8 kcal/mol) and formed the highest number of binding interactions (14), suggesting a superior inhibitory potential compared to the other compounds (Table 4).

The enhanced binding affinity of derivative **4** appears to result from its capacity to establish multiple electrostatic interactions. Specifically, the π-electrons of its indole ring formed a double π–cation interaction with the guanidinium group of Arg120, as well as a π–anion interaction with the carboxylate group of Glu524. Additional stability was conferred through hydrophobic contacts and a π–sulfur interaction between the benzene ring of compound **4** and the sulfur atom of Met522. A similar binding mode was observed for derivative **3**, the other aromatic amino acid derivative. However, its slightly less favorable docking score may be attributed to its inability to establish the aforementioned electrostatic interactions. In contrast, dexketoprofen amide derivatives containing aliphatic amino acids exhibited higher docking scores and formed significantly fewer binding contacts (Figure 9). Derivatives **1** and **5**, due to their high degree of structural similarity, exhibited nearly identical binding modes within the active site of COX-2. Their interactions were primarily characterized by the formation of two conventional hydrogen bonds with the Arg120 residue and an additional hydrogen bond with Tyr355, both of which function as hydrogen bond donors. In contrast, derivative **2** formed two conventional hydrogen bonds with Met522 (hydrogen bond acceptor) and Ala527 (hydrogen bond donor), indicating a distinct binding pattern.

The reduced docking score values observed for the aliphatic derivatives, in comparison to the aromatic amino acid derivatives, are likely attributable to their limited capacity to establish extensive electrostatic interactions. Notably, all aliphatic derivatives engaged in a π–cation interaction with Arg120. However, derivative **1** also exhibited an additional π–anion interaction with Glu524, potentially enhancing its binding affinity. Overall, the aliphatic derivatives demonstrated a significantly lower total number of stabilizing interactions relative to the aromatic derivatives, which may account for the reduced stability of their respective ligand–enzyme complexes. At the same time, all newly synthesized compounds exhibited lower docking scores when binding to the COX-2 active site compared to COX-1. This observation can be attributed to the larger and more spacious nature of the COX-2 binding pocket, into which newly synthesized dexketoprofen derivatives fit more easily. In contrast, the comparatively narrower active site of COX-1 likely impedes optimal ligand fitting, thereby reducing binding affinity [68,69].

These findings suggest that structural modifications of dexketoprofen introducing aromatic moieties facilitate the formation of bulkier molecules and supplementary electrostatic and hydrophobic interactions, thereby enhancing the binding affinity of the compounds toward COX-2.

Previously published studies have demonstrated that ketoprofen amide derivatives exhibit a higher binding affinity for COX-2 compared to COX-1 [41,59]. Specifically, structural analyses revealed that when the co-crystallized ligand ibuprofen is fitted into the active site of COX-1, it forms three key conventional hydrogen bonds via its carboxylate group: two with the Arg120 residue and one with Tyr355. Ketoprofen, which maintains the ability to engage in these hydrogen-bonding interactions, demonstrated a similar binding mode and comparable docking score to ibuprofen (−9.53 kcal/mol for ketoprofen vs. −9.22 kcal/mol for ibuprofen). In contrast, the ketoprofen amide derivatives evaluated in the same study were unable to establish these critical interactions, resulting in significantly less favorable docking scores relative to ibuprofen. Furthermore, the capacity of the ketoprofen amide derivatives to form key hydrogen bonds within the COX-2 active site contributed to their enhanced selectivity toward the COX-2 isoform [59]. These computational findings are partially consistent with those from in vitro assays. Specifically, dexketoprofen demonstrated a higher potential for COX-1 inhibition compared to the newly synthesized compounds. However, the in silico docking results for dexketoprofen within the COX-2 active site did not align with the in vitro findings, where dexketoprofen showed markedly superior COX-2 inhibition in comparison to the newly synthesized compounds. Conversely, among the tested derivatives, compound **4** exhibited the greatest COX-2 inhibitory activity and was the only newly synthesized molecule for which an IC_50_ value could be determined.

## 4. Conclusions

In the present study, five novel amide derivatives of dexketoprofen were synthesized with the aim of enhancing anti-inflammatory and antioxidant activities. The synthetic approach involved the functionalization of the carboxyl group and the extension of the spacer between the carboxyl group and the aromatic moiety of dexketoprofen.

Among the analyzed compounds, dexketoprofen alanine and tryptophan derivatives (compounds **2** and **4**) exhibited the most pronounced in vivo anti-inflammatory activity, as indicated by the highest percentage of rat paw edema inhibition at the fourth hour after inflammation induction. These effects were comparable to those observed for the parent compound dexketoprofen. Furthermore, in vitro obtained results revealed weak inhibitory activity towards COX-1 for all novel compounds, while only the tryptophan derivative (compound **4**) demonstrated the significant inhibition of COX-2, suggesting that amide formation in some cases might promote the selective COX-2 inhibition, especially if the aromatic amino acid residue is present within its side chain. The high binding affinity of derivative **4** for COX-2 can be further elaborated by its capacity to establish multiple electrostatic interactions with the active site of COX-2. In terms of antioxidant activity, dexketoprofen alanine and GABA derivatives (compounds **2** and **5**) showed comparable efficacy to dexketoprofen in restoring redox balance, suggesting their significant antioxidant capacity in conditions of acute inflammation.

Our findings highlight the therapeutic potential of novel dexketoprofen amide derivatives as dual-function agents capable of modulating both inflammatory processes and oxidative stress. This dual mechanism of action, reflected by suppression of both inflammation and oxidative stress, may offer improved efficacy in treating inflammatory conditions characterized by oxidative imbalance. Among the tested compounds, derivative **2** stands out as the most promising drug candidate due to its notable anti-inflammatory and antioxidant activities. Despite its promising profile, further research is required to clarify the exact molecular mechanisms underlying these effects.

## Figures and Tables

**Table 1:** Results of relative organ weights.

Compound	1	2	3	4	5	Control Group
Organ						
Kidney	0.35 ± 0.07	0.39 ± 0.05	0.36 ± 0.04	0.37 ± 0.04	0.36 ± 0.06	0.38 ± 0.02
Heart	0.33 ± 0.03	0.36 ± 0.04	0.34 ± 0.02	0.33 ± 0.04	0.34 ± 0.02	0.35 ± 0.03
Liver	2.84 ± 0.20	3.15 ± 0.50	2.98 ± 0.23	2.67 ± 0.14	3.03 ± 0.41	3.05 ± 0.12
Stomach	0.57 ± 0.07	0.61 ± 0.05	0.55 ± 0.04	0.57 ± 0.06	0.58 ± 0.05	0.59 ± 0.07

**Table 2:** Anti-inflammatory activity of dexketoprofen derivatives in a carrageenan-induced rat paw edema model.

Rat Paw Thickness (mm) (% of Inhibition)					
Experimental Groups	0 h	1 h	2 h	3 h	4 h
Compound **1**	3.58 ± 0.13	5.68 ± 0.25 (5.263%)	5.58 ± 0.34 (13.669%)	5.16 ± 0.34 (16.842%)	4.50 ± 0.29 (18.824%)
Compound **2**	4.66 ± 0.13	6.52 ± 0.28 (16.090%)	6.36 ± 0.25 (26.619%) *	5.92 ± 0.19 (33.684%) *	5.08 ± 0.54 (62.941%) **
Compound **3**	4.27 ± 0.21	6.25 ± 0.25 (10.526%)	6.10 ± 0.18 (20.863%) *	5.73 ± 0.23 (22.807%)	4.93 ± 0.37 (41.176%)
Compound **4**	4.02 ± 0.12	5.90 ± 0.37 (15.038%)	5.77 ± 0.36 (24.460%) *	5.38 ± 0.34 (28.070%) *	4.48 ± 0.33 (58.824%) **
Compound **5**	3.46 ± 0.29	5.52 ± 0.25 (7.068%)	5.38 ± 0.48 (17.122%)	5.00 ± 0.29 (18.947%)	4.28 ± 0.37 (27.647%) *
Solvent	3.68 ± 0.41	5.90 ± 0.24	6.00 ± 0.40	5.58 ± 0.37	4.82 ± 0.23
Dexketoprofen	4.10 ± 0.31	5.93 ± 0.08 (17.293%)	5.78 ± 0.15 (27.338%) *	5.38 ± 0.23 (32.456%) *	4.53 ± 0.23 (61.765%) **

The results are presented as mean value ± standard deviation (SD). * A statistically significant difference at the level of *p* < 0.05 in relation to the solvent group. ** A statistically significant difference at the level of *p* < 0.01 in relation to the solvent group.

**Table 3:** Results of COX-1 and COX-2 inhibition activity presented as IC_50_ values (μM).

Compound	COX-1	COX-2
**1**	>100	>100
**2**	>100	>100
**3**	>100	>100
**4**	>100	37.55 ± 3.43
**5**	>100	>100
Dexketoprofen	0.0015 ± 0.0006	0.13 ± 0.02

**Table 4:** Molecular docking results of dexketoprofen and its amide derivatives in interaction with COX-1 and COX-2.

Compound	COX-1		COX-2	
	Docking Score	Interacting Residue *	Docking Score	Interacting Residue *
1	−8.2	**Arg120** (HBD × 3), Val349 (π-σ), Leu352 (π-σ), Tyr355 (bump), Ile523 (π-alkyl), Ala527 (π-alkyl × 2), **Ser530** (HBD)	−9.0	Val89 (π-σ), Leu93 (π-σ × 2), Val116 (π-alkyl), **Arg120** (HBD × 2), Arg120 (π-cation), **Tyr355** (HBD), Glu524 (π-anion)
2	−7.6	Leu93 (π-alkyl), Leu115 (π-alkyl), Val116 (π-σ × 3), Val119 (π-alkyl), **Arg120** (HBD × 2), Tyr355 (π-π), **Ile523** (HBA)	−9.4	Val116 (π-σ), Arg120 (π-cation), Val349 (π-alkyl), Tyr355 (π-π), **Met522** (HBA), **Ala527** (HBD), Ala527 (π-alkyl)
3	−8.8	**Arg97** (HBD × 4), Gln192 (CHBD), **Tyr355** (HBA)	−9.9	Val89 (π-alkyl), **Arg120** (HBD), Arg120 (π-cation), Val349 (π-σ), Leu352 (π-alkyl), **Tyr355** (HBD), Met522 (π-sulfur), Val523 (π-alkyl), Gly526 (amide-π), Ala527 (π-σ), Leu531 (π-alkyl)
4	−8.8	**Arg97** (HBD × 2), Gln192 (CHBD), **Gln350** (HBD)	−10.8	Val89 (π-alkyl), Val89 (π-σ), **Arg120** (HBD), Arg120 (π-cation × 2), Val349 (π-σ), Leu352 (π-alkyl), **Tyr355** (HBD), Met522 (π-sulfur), Val523 (π-alkyl), Glu524 (π-anion), Gly526 (amide-π), Ala527 (π-σ), Leu531 (π-alkyl)
5	−8.5	**Arg120** (HBD), Val349 (π-σ), Leu352 (π-σ), Ile523 (π-alkyl), **Glu524** (HBA), Gly526 (amide-π), Ala527 (π-alkyl), **Ser530** (HBD)	−9.2	Val89 (π-σ), Leu93 (π-alkyl), Val116 (π-σ), **Arg120** (HBD × 2), Arg120 (π-cation), **Tyr355** (HBD), Tyr355 (π-π), Val523 (CHBD)
Dexketoprofen	−8.9	Arg120 (HBD × 2), Val349 (π-σ), Leu352 (π-alkyl × 2), Ile523 (π-alkyl), Gly526 (amide-π), Ala527 (π-alkyl), Ser530 (HBD)	−8.9	Tyr385 (HBD), Ser530 (HBD), Ser530 (CHBD), Val349 (π-alkyl), Leu352 (π-alkyl), Val523 (π-alkyl), Ala527 (π-alkyl × 2), Val116 (π-alkyl), Leu359 (π-alkyl), Leu531 (π-alkyl)

* Residues engaged in conventional hydrogen bonding are denoted in bold. HBD—hydrogen bond donor, HBA—hydrogen bond acceptor, CHBD—carbon–hydrogen bond donor, bump—unfavorable binding interaction.

## Data Availability

Data are contained within the article.

## Supplementary Materials

The following supporting information can be downloaded at: https://www.mdpi.com/article/10.3390/antiox14070796/s1, Figure S1: ^1^H NMR spectrum (a) and ^13^C NMR (b) of compound **1**; Figure S2: ^1^H NMR spectrum (a) and ^13^C NMR (b) of compound **2**; Figure S3: ^1^H NMR spectrum (a) and ^13^C NMR (b) of compound **3**; Figure S4: ^1^H NMR spectrum (a) and ^13^C NMR (b) of compound **4**; Figure S5: ^1^H NMR spectrum (a) and ^13^C NMR (b) of compound **5**.
