# Supplementary material for: From Chemistry to Pharmacology: Exploring the Anti-Inflammatory and Antioxidant Potential of Novel Dexketoprofen Amide Derivatives
Source: Antioxidants (Basel). 2025 Jun 27;14(7):796. doi: 10.3390/antiox14070796 (PMC12291762; doi:10.3390/antiox14070796)
Supplement: Supplementary file 1 [file antioxidants-14-00796-s001.zip › antioxidants-3700021-supplementary.pdf]

ANTIOXIDANTS

Supplementary Information associated with the paper

From Chemistry to Pharmacology: Exploring the Anti-Inflammatory and Antioxidant Potential of Novel Dexketoprofen Amide Derivatives

Marko Karović [1], Miloš Nikolić [1,*], Nikola Nedeljković [1], Marina Vesović [1], Marina Nikolić [2,3], Marijana Anđić [1,3], Nevena Lazarević [1,3,4], Vladimir Jakovljević [2,3,4], Jelena Nedeljković [5,6], Sanja Đaković [1], Jelena Bošković [7] and Vladimir Dobričić [7]

[1] Department of Pharmacy, Faculty of Medical Sciences, University of Kragujevac, Serbia; karovic.marko.kg@gmail.com (M.K.); nikola.nedeljkovic@fmn.kg.ac.rs (N.N.), marina.vesovic@fmn.kg.ac.rs (M.V.); andjicmarijana10@gmail.com (M.A.); nevena.lazarevic@fmn.kg.ac.rs (N.L.); sanjadjakovic1996@gmail.com (S.Đ.)

[2] Department of Physiology, Faculty of Medical Sciences, University of Kragujevac, Serbia; marina.rankovic.95@gmail.com (M.N.); drvladakgbg@yahoo.com (V.J.)

[3] Center of Excellence for Redox Balance Research in Cardiovascular and Metabolic Disorders, Kragujevac, Serbia

[4] Department of Human Pathology, 1st Moscow State Medical University IM Sechenov, Moscow, Russia

[5] Department of Medical statistics and informatics, Faculty of Medical Sciences, University of Kragujevac, 34000 Kragujevac, Serbia; jelena.dimitrijevic10@gmail.com (J.N.)

[6] Center for Molecular Medicine and Stem Cell Research, Faculty of Medical Sciences, University of Kragujevac, Serbia

[7] Department of Pharmaceutical Chemistry, Faculty of Pharmacy, University of Belgrade, 11221 Belgrade, Serbia; jelena.boskovic@pharmacy.bg.ac.rs (J.B.); vladimir.dobricic@pharmacy.bg.ac.rs (V.D.)

* Correspondence: milos.nikolic@fmn.kg.ac.rs

Contents

1. NMR spectra of compounds 1-5.

^{1}H NMR spectrum (a) and ^{13}C NMR (b) of compound **1** (DKGLI). 3

^{1}H NMR spectrum (a) and ^{13}C NMR (b) of compound **2** (DKALA). 4

^{1}H NMR spectrum (a) and ^{13}C NMR (b) of compound **3** (DKPHE). 5

^{1}H NMR spectrum (a) and ^{13}C NMR (b) of compound **4** (DKTRP). 6

^{1}H NMR spectrum (a) and ^{13}C NMR (b) of compound **5** (DKGABA). 7

a)

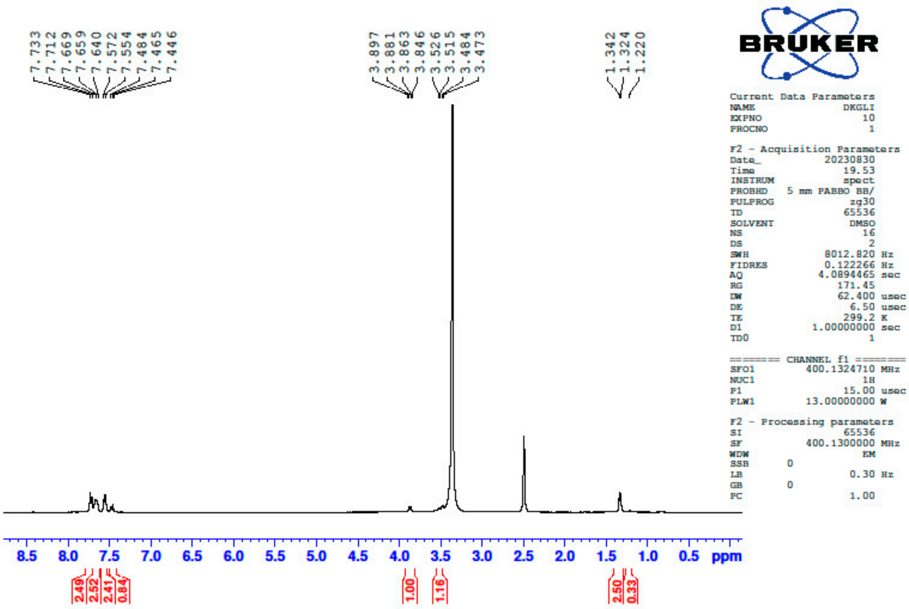

b)

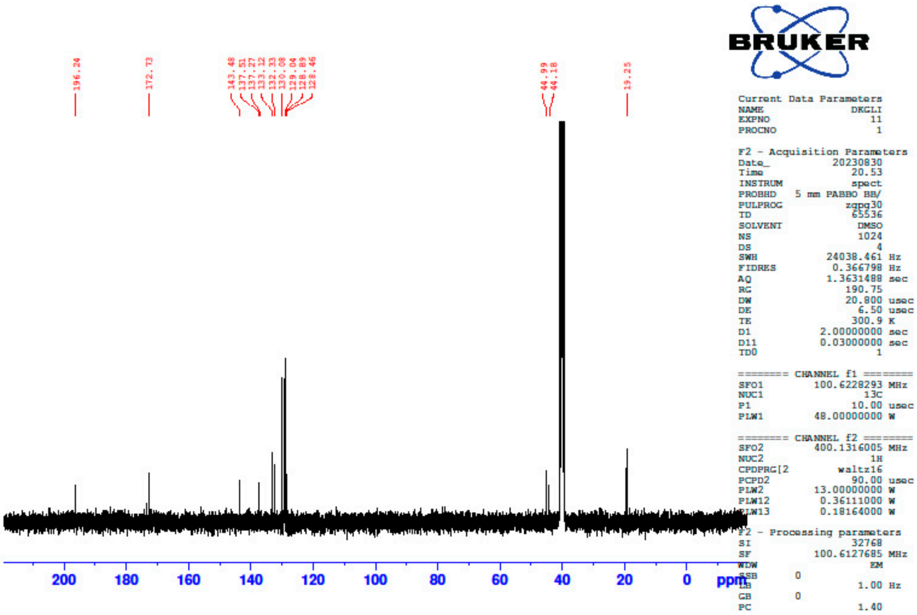

Figure S1. ^{1}H NMR spectrum (a) and ^{13}C NMR (b) of compound **1 (DKGLI)**.

a)

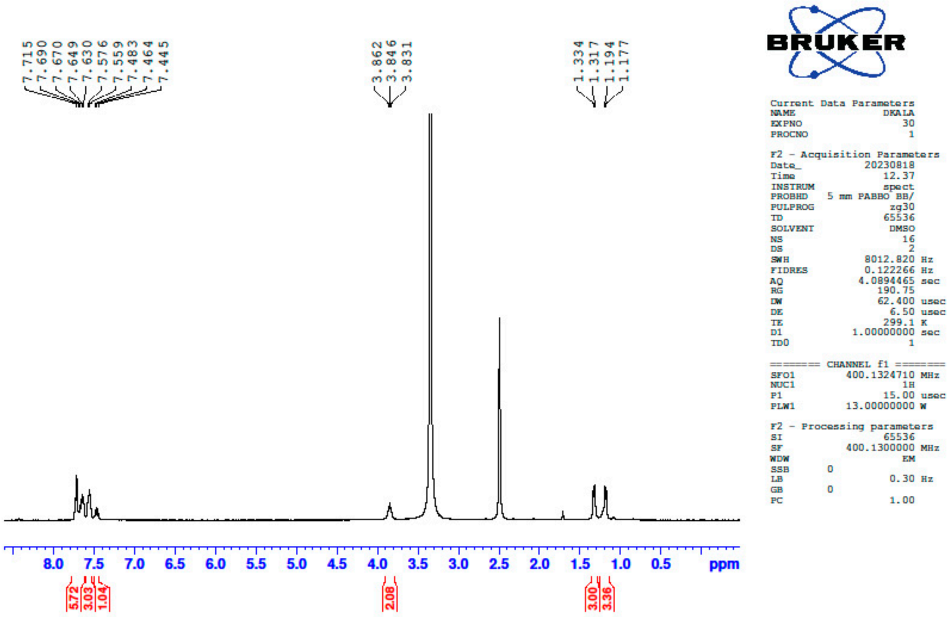

b)

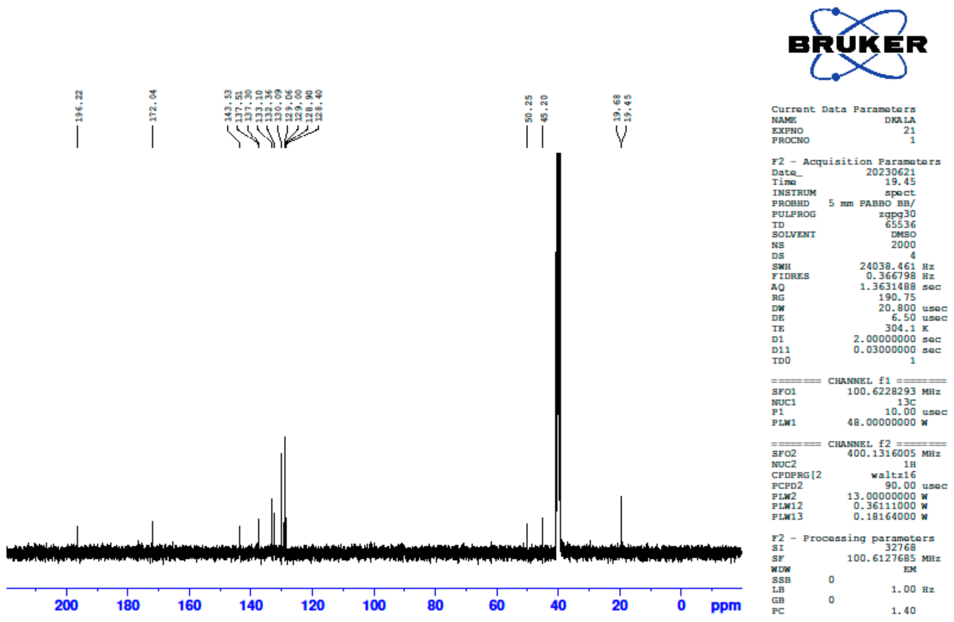

Figure S2. ^{1}H NMR spectrum (a) and ^{13}C NMR (b) of compound **2** (**DKALA**).

a)

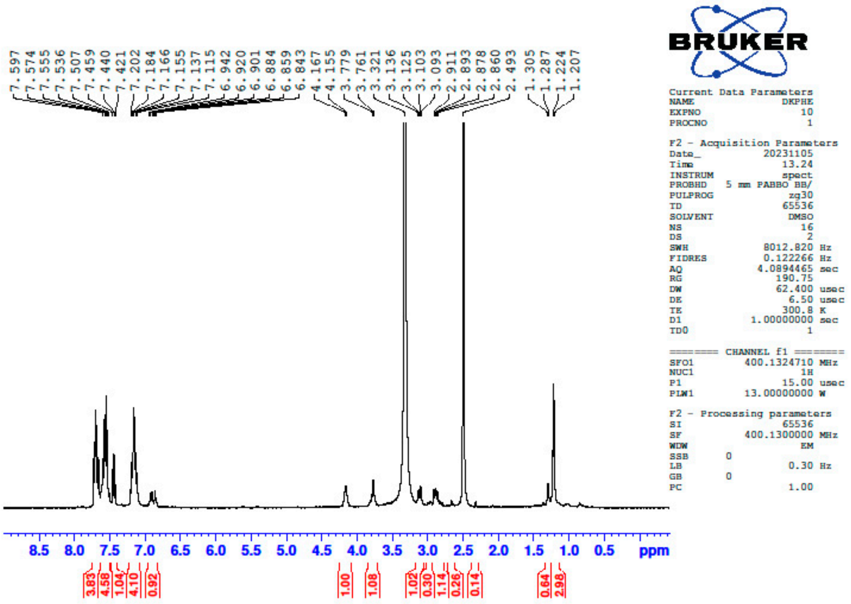

b)

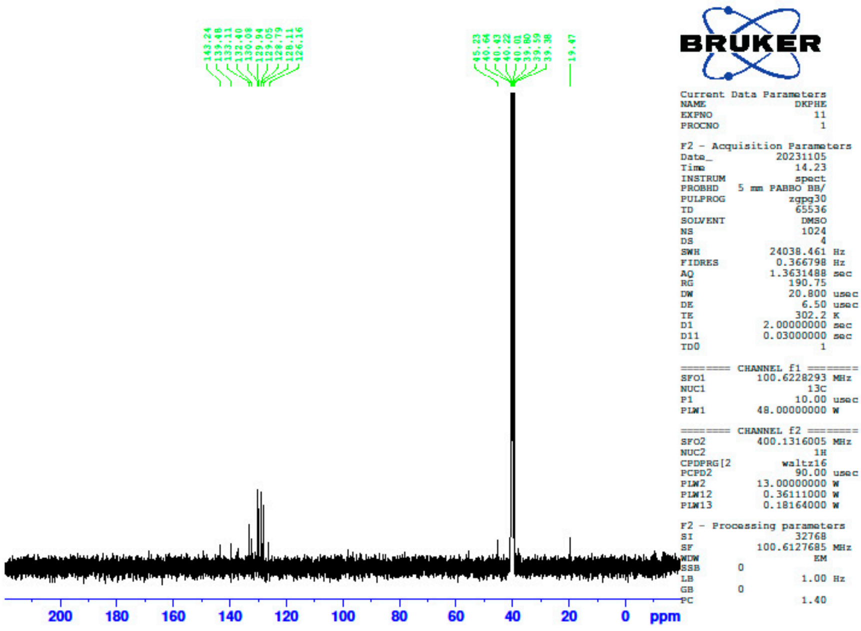

Figure S3. ^{1}H NMR spectrum (a) and ^{13}C NMR (b) of compound **3** (**DKPHE**).

a)

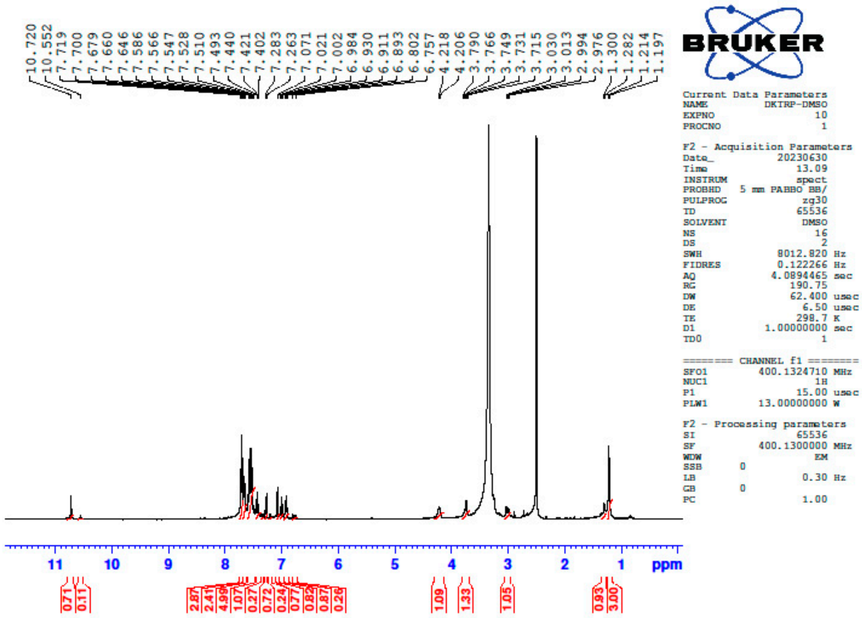

b)

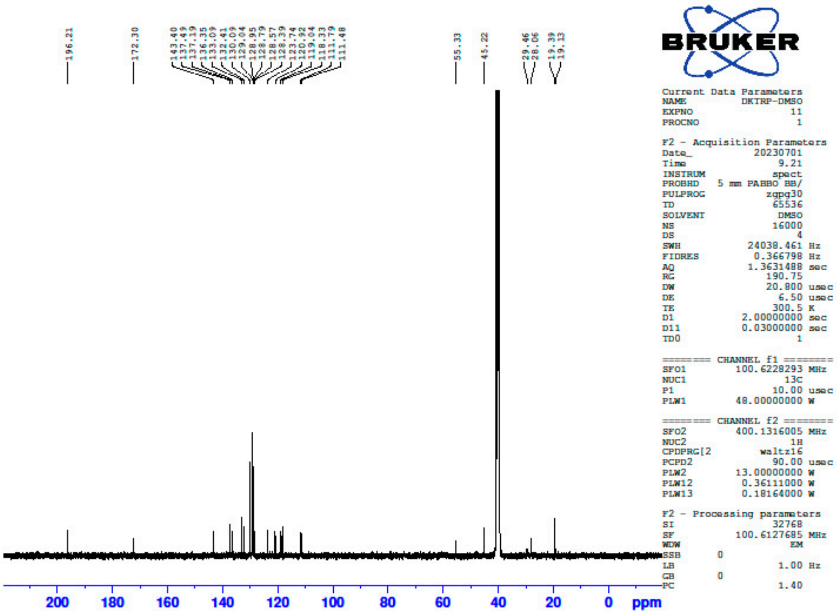

Figure S4. ^{1}H NMR spectrum (a) and ^{13}C NMR (b) of compound **4** (**DKTRP**).

a)

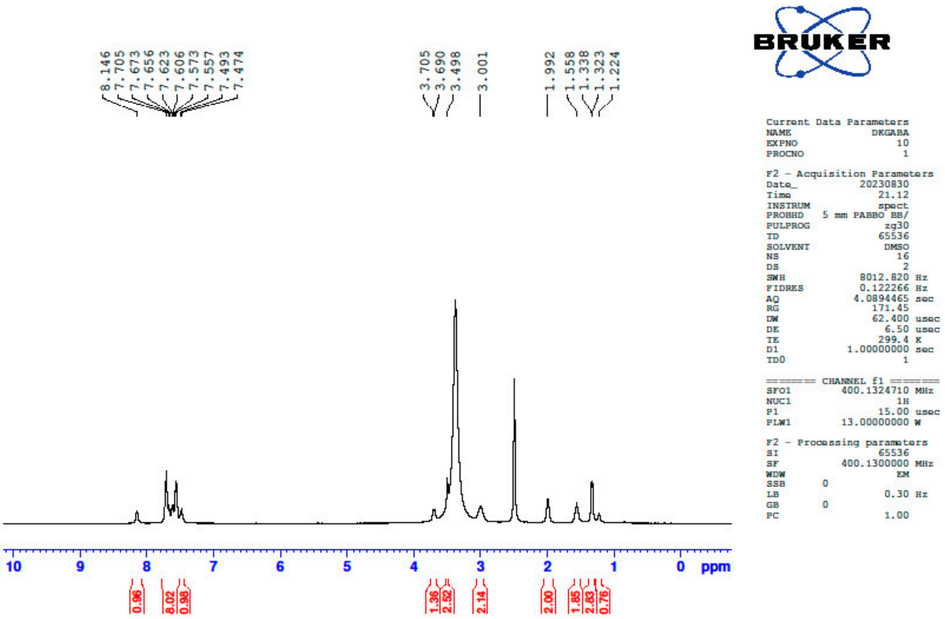

b)

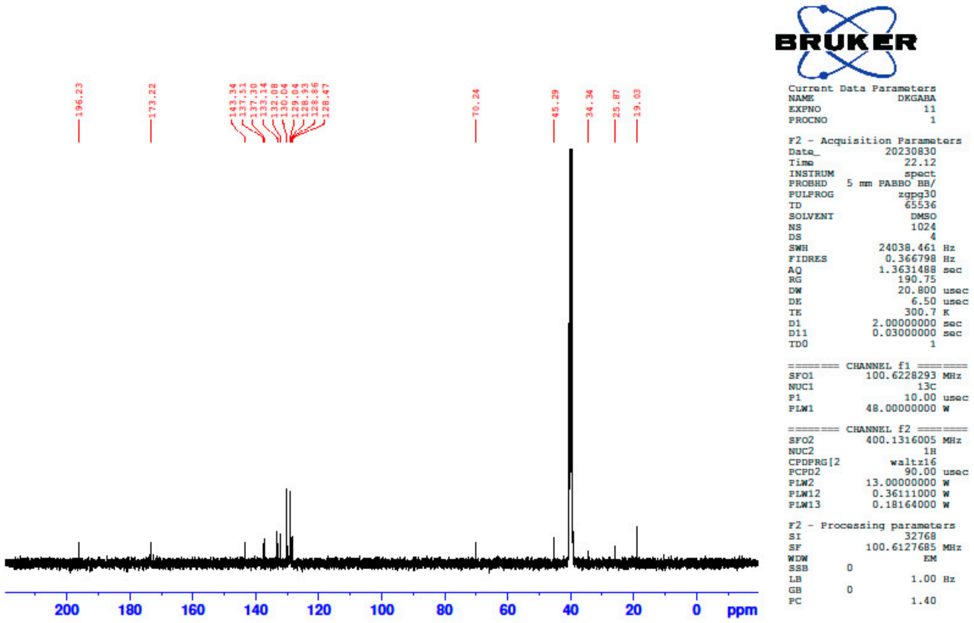

Figure S5. ^{1}H NMR spectrum (a) and ^{13}C NMR (b) of compound **5** (**DKGABA**).
